# Role of Plasmodium falciparum Protein GEXP07 in Maurer’s Cleft Morphology, Knob Architecture, and P. falciparum EMP1 Trafficking

**DOI:** 10.1128/mBio.03320-19

**Published:** 2020-03-17

**Authors:** Emma McHugh, Olivia M. S. Carmo, Adam Blanch, Oliver Looker, Boyin Liu, Snigdha Tiash, Dean Andrew, Steven Batinovic, Andy J. Y. Low, Hyun-Jung Cho, Paul McMillan, Leann Tilley, Matthew W. A. Dixon

**Affiliations:** a Department of Biochemistry and Molecular Biology, Bio21 Molecular Science and Biotechnology Institute, University of Melbourne, Parkville, VIC, Australia; b Biological Optical Microscopy Platform, Bio21 Molecular Science and Biotechnology Institute, University of Melbourne, Parkville, VIC, Australia; National Institutes of Health

**Keywords:** malaria, protein trafficking, virulence determinants

## Abstract

The malaria parasite Plasmodium falciparum traffics the virulence protein P. falciparum erythrocyte membrane protein 1 (*Pf*EMP1) to the surface of infected red blood cells (RBCs) via membranous organelles, known as the Maurer’s clefts. We developed a method for efficient enrichment of Maurer’s clefts and profiled the protein composition of this trafficking organelle. We identified 13 previously uncharacterized or poorly characterized Maurer’s cleft proteins. We generated transfectants expressing green fluorescent protein (GFP) fusions of 7 proteins and confirmed their Maurer’s cleft location. Using co-immunoprecipitation and mass spectrometry, we generated an interaction map of proteins at the Maurer’s clefts. We identified two key clusters that may function in the loading and unloading of *Pf*EMP1 into and out of the Maurer’s clefts. We focus on a putative *Pf*EMP1 loading complex that includes the protein GEXP07/CX3CL1-binding protein 2 (CBP2). Disruption of GEXP07 causes Maurer’s cleft fragmentation, aberrant knobs, ablation of *Pf*EMP1 surface expression, and loss of the *Pf*EMP1-mediated adhesion. ΔGEXP07 parasites have a growth advantage compared to wild-type parasites, and the infected RBCs are more deformable and more osmotically fragile.

## Abstract (precis)

The trafficking of the virulence antigen *Pf*EMP1 and its presentation at the knob structures at the surface of parasite-infected RBCs are central to severe adhesion-related pathologies such as cerebral and placental malaria. This work adds to our understanding of how PfEMP1 is trafficked to the RBC membrane by defining the protein-protein interaction networks that function at the Maurer’s clefts controlling PfEMP1 loading and unloading. We characterize a protein needed for virulence protein trafficking and provide new insights into the mechanisms for host cell remodeling, parasite survival within the host, and virulence.

## INTRODUCTION

Each year, Plasmodium falciparum causes ∼200 million cases of illness in humans and more than 400,000 deaths, mostly of children under the age of 5 years (1). In the asexual blood stage of infection, parasites invade red blood cells (RBCs) and develop through the so-called ring, trophozoite (growing), and schizont (dividing) stages, eventually releasing invasive merozoites that continue the blood cycle. During this cycle, the parasite induces marked changes to the host RBC, including the elaboration of new organelles in the RBC cytoplasm, known as the Maurer’s clefts (MC), and the establishment of protrusions at the RBC membrane, known as knobs. The knobs comprise a spiral protein structure supported by a knob-associated histidine-rich protein (KAHRP) (2). The knob acts as a scaffold for the presentation of the major virulence antigen P. falciparum erythrocyte membrane protein 1 (*Pf*EMP1). This virulence complex has an important role in adhesion of infected RBCs to endothelial cell receptors. Rigid mature-stage-infected RBCs are sequestered away from the circulation, thus avoiding recognition and removal during passage through the spleen (3). *Pf*EMP1 is exported into the host RBC cytoplasm via a translocon at the parasitophorous vacuole membrane (4, 5). The current model suggests that *Pf*EMP1 is trafficked to Maurer’s clefts as a soluble, chaperoned complex (6, 7), where it is inserted into the membrane bilayer (8) before repackaging for vesicle-mediated trafficking to the RBC surface (9).

Maurer’s clefts are roughly disc-shaped cisternae, with a diameter of ∼500 nm (10). In the ring stage (up to ∼20 h post invasion), they are mobile in the RBC cytoplasm but later become tethered to the RBC membrane skeleton (11). Remodeled host actin filaments and parasite-derived tethers connect the Maurer’s clefts to the RBC membrane (12, 13). However, the precise role of the Maurer’s clefts in protein trafficking remains unclear, and their composition has been defined only partly (14). In particular, there is very limited information about interactions between proteins of the Maurer’s clefts and their virulence-associated cargo (15, 16).

In this work, we developed a method for enriching “mobile” Maurer’s clefts from parasites at 14 to 18 h post invasion. We provide a detailed analysis of the Maurer’s cleft proteome, identifying and validating several novel resident proteins. Using super resolution optical microscopy, we defined the spatial organization of new and established Maurer’s cleft proteins. We used co-immunoprecipitation (co-IP) and mass spectrometry (MS) to describe a network of protein interactions, identifying two *Pf*EMP1-interacting complexes, one of which we propose is responsible for loading *Pf*EMP1 into the Maurer’s clefts. We show that deletion of a partially characterized Maurer’s cleft component, *PF*3D7_1301700 (GEXP07/CX3CL1-binding protein 2 [CBP2]), leads to defective *Pf*EMP1 trafficking, altered Maurer’s cleft architecture, aberrant knob formation, and a loss of parasite adhesion to endothelial ligands. These changes are accompanied by decreased cellular rigidity, increased osmotic fragility, and a marked growth advantage.

## RESULTS

### Enrichment of Maurer’s clefts from infected RBCs.

**FIG 1:**
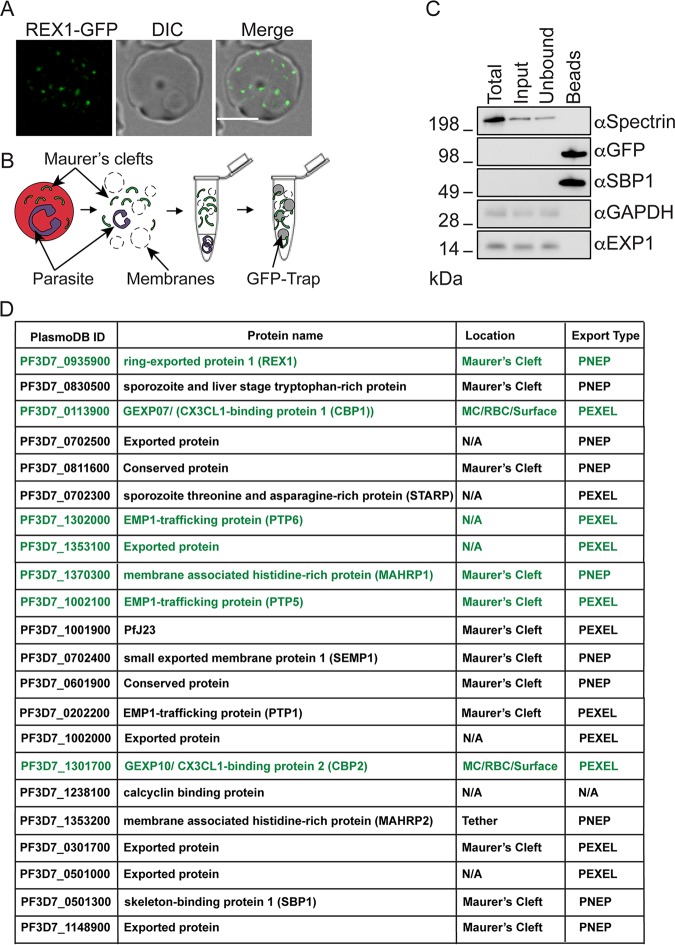
Enrichment and analysis of Maurer’s clefts from P. falciparum REX1-GFP-infected RBCs. (A) Live-cell fluorescence of an RBC infected with a REX1-GFP-expressing parasite at 14 to 18 h post invasion. GFP (green), differential interference contrast (DIC), and merged images are shown. Scale bar 5 μm. (B) Schematic overview of the GFP-Trap Maurer’s cleft enrichment method. Infected RBCs (14 to 18 h post invasion) are hypotonically lysed and subjected to differential centrifugation, and then the REX1-GFP-labeled Maurer’s clefts are captured with GFP-Trap beads. (C) Western blotting to assess enrichment of Maurer’s clefts from REX1-GFP-infected RBCs. Due to the high levels of hemoglobin in the total, input, and unbound fractions, it was necessary to dilute these. These are 1% equivalents with the IP representing 100%. Total = complete infected RBC lysate; Input = fraction applied to the GFP-Trap beads; Unbound = supernatant after GFP-Trap bead incubation; Beads = material bound to beads. Molecular masses are in kilodaltons. (D) Summary of proteins identified in the mass spectrometric analysis of enriched REX1-GFP Maurer’s clefts. See Data Set 1 at https://doi.org/10.26188/5e4f43123b214 for more detail. Proteins chosen for GFP tagging are indicated in green text. MC = Maurer’s clefts; RBC = red blood cell; Surface = red blood cell surface; N/A = not available; PEXEL = *Plasmodium* export element; PNEP = PEXEL-negative exported protein.

Maurer’s clefts are parasite-derived membrane-bound structures that remain mobile within the RBC cytoplasm until ∼20 h post invasion (see Movie S1 in the supplemental material). Leveraging this biology, we developed a protocol for purifying Maurer’s clefts from parasite-infected RBCs at 14 to 18 h post invasion. Fluorescence microscopy of transfectants expressing green fluorescent protein (GFP)-tagged ring exported protein 1 (REX1-GFP [17]) confirmed a typical Maurer’s cleft profile (Fig. 1A). REX1-GFP transfectant-infected RBCs were hypotonically lysed, and the suspension was extruded through a 27-gauge needle, adjusted to isotonicity, precleared, and incubated with GFP-Trap beads (Fig. 1B). Fluorescence microscopy of the beads (see Fig. S1A in the supplemental material) confirms the presence of GFP-containing structures with dimensions consistent with a mixture of intact (∼500-nm) and fragmented Maurer’s clefts. Fractions from the total cell lysate, the cleared input, and the unbound and bound fractions were analyzed by Western blotting (Fig. 1C; full-length blots are shown in Fig. S1B). Due to the high hemoglobin content in the total, input, and unbound fractions, the samples were diluted (1% equivalent relative to the bead fraction) before electrophoresis; as a result, REX1-GFP and SBP1 were not detected in these fractions. REX1-GFP and the known Maurer’s cleft protein skeleton-binding protein 1 (SBP1 [18, 19]) were found to be highly enriched in the bound fraction, while spectrin, a component of the host cytoskeleton, was not detected, consistent with a high level of enrichment. The parasitophorous vacuole membrane (PVM) protein exported protein-1 (EXP1) and the parasite cytoplasmic protein P. falciparum glyceraldehyde-3 phosphate dehydrogenase (*Pf*GAPDH) were also found to be absent (Fig. 1C).

10.1128/mBio.03320-19.1 FIG S1 Live-cell microscopy of REX1-GFP bound to GFP-Trap beads and full-length Western blots of GFP-tagged transfectants. (A) Fluorescence microscopy of the beads reveals structures with dimensions consistent with a mixture of intact (∼500-nm) and fragmented Maurer’s clefts. Scale bar = 5 μm. (B) Full uncropped Western blot images for assessing enrichment of Maurer’s clefts from REX1-GFP-infected RBCs. These blots were cut horizontally in some cases to simultaneously probe for proteins of different sizes. Total = complete infected RBC lysate; Input = fraction applied to GFP-Trap beads; Unbound = supernatant after GFP-Trap bead incubation, Beads = material bound to beads. Molecular masses are in kilodaltons. (C) Western blot of saponin-lysed infected RBCs from GFP-tagged transfectant parasite lines. Green arrowheads indicate predicted size of the mature full-length protein. Molecular masses are in kilodaltons. Download FIG S1, PDF file, 0.1 MB. Copyright © 2020 McHugh et al. 2020 McHugh et al. This content is distributed under the terms of the Creative Commons Attribution 4.0 International license.

10.1128/mBio.03320-19.8 MOVIE S1 MC mobility movie. The movie shows the mobility of the Maurer’s clefts (green) in 14-to-18-h-post invasion parasite-infected RBCs. Download Movie S1, AVI file, 0.04 MB. Copyright © 2020 McHugh et al. 2020 McHugh et al. This content is distributed under the terms of the Creative Commons Attribution 4.0 International license.

### Mass spectrometry analysis of the enriched Maurer’s clefts.

The captured Maurer’s clefts were released from the beads, digested with trypsin, and analyzed by liquid chromatography-tandem mass spectrometry (LC-MS/MS). Proteins were deemed significant if two or more peptides were detected in the Maurer’s cleft (REX1-GFP) sample and were 3-fold enriched compared to the 3D7 control, in 2 separate experiments (Fig. 1D; see also Data Set 1 at https://doi.org/10.26188/5e4f43123b214). Five well-characterized Maurer’s cleft proteins were identified, namely, REX1 (20), *Pf*EMP1 trafficking protein 1 (PTP1 [21]), skeleton binding protein 1 (SBP1 [22]), small exported membrane protein 1 (SEMP1 [15]), and membrane-associated histidine-rich protein 1 (MAHRP1 [23]). A tether protein, membrane-associated histidine-rich protein 2 (MAHRP2 [13]), was also identified.

Six partially characterized proteins that have been reported previously to be Maurer’s cleft-located proteins were identified, namely, parasite-infected erythrocyte surface protein 2 (PIESP2 [24]); sporozoite- and liver-stage tryptophan-rich protein (25); *Pf*J23 (14); and *PF*3D7_0301700, *PF*3D7_1148900, *PF*3D7_0702500, and *PF*3D7_0601900 (15, 25, 26).

We identified *PF*3D7_1301700 and *PF*3D7_0113900, for which there are conflicting reports with respect to cellular location, ranging from the RBC cytosol in gametocytes (27) to the Maurer’s clefts in asexual stages (28) to the RBC surface (29). These proteins have been previously referred to as gametocyte exported protein 7 (GEXP07)/CX3CL1-binding protein 2 (CBP2) and GEXP10/CBP1 (27, 29). Here, we refer to these proteins as GEXP07 and GEXP10.

A further 7 proteins were identified for which no location data have been reported previously. All but one protein (calcyclin-binding protein) has a predicted secretory signal or transmembrane segment, and 5 have a *Plasmodium* export element (PEXEL) motif, which predicts export of parasite proteins to the host RBC (30). The following four of these proteins have been successfully genetically disrupted: PTP5, PTP6, *PF*3D7_1353100, and *PF*3D7_0501000 (21). The remaining proteins, *PF*3D7_0702300, *PF*3D7_0811600, *PF*3D7_1002000, *PF*3D7_1035800, and sporozoite threonine and asparagine-rich protein (STARP), do not have characterized locations. Over 100 peptides were observed for *Pf*EMP1 itself (Data Set 1), consistent with a number of studies showing that *Pf*EMP1 is highly enriched at the Maurer’s clefts (8, 31, 32), with only a subpopulation reaching the RBC membrane. Some RBC proteins were also identified in the enriched Maurer’s clefts, including annexin A4 and A11 and copine-3, which are calcium-dependent phospholipid-binding proteins involved in membrane remodeling (33), as well as VPS28, TSG101, syntaxin, and VAC14—proteins that are involved in membrane binding, vesicle trafficking, and lipid biogenesis (see Table S1 at https://doi.org/10.26188/5e4f42d4c65fe; see also Data Set 1).

### GFP tagging of proteins confirms their Maurer’s cleft location.

**FIG 2:**
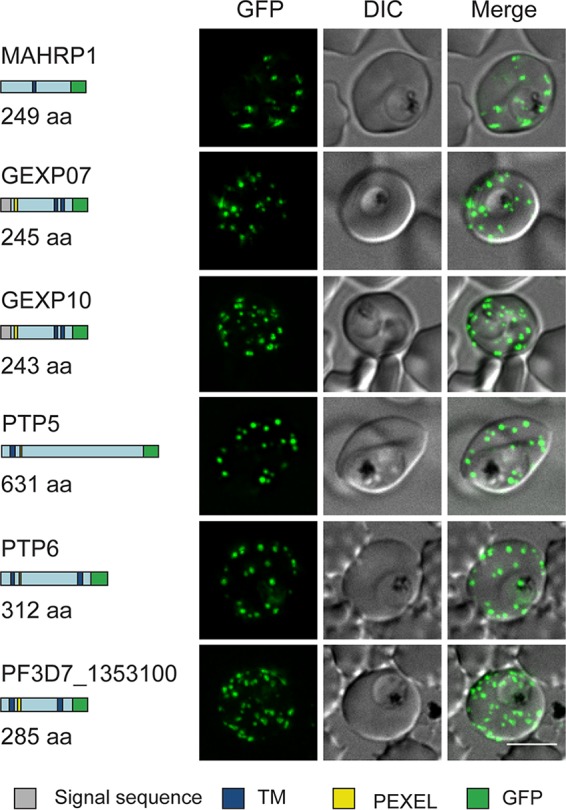
Live-cell fluorescence analysis of GFP-tagged exported proteins. Predicted native protein lengths (amino acids [aa]) and schematic representations of six proteins that were selected for GFP tagging are shown on the left. Gray = signal sequence, blue = transmembrane domain (TM), yellow = PEXEL motif, green = GFP tag. Live-cell fluorescence and DIC microscopy of transfectants expressing GFP-tagged putative Maurer’s cleft proteins revealed fluorescent puncta in the RBC cytoplasm. Scale bar = 5 μm.

**FIG 3:**
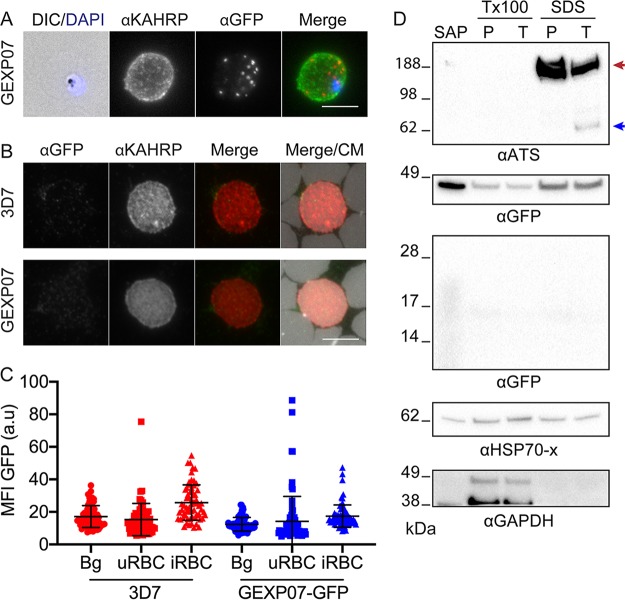
GEXP07-GFP is not detectable at the infected RBC membrane. (A) Whole-cell immunofluorescence of GEXP07-GFP parasites labeled with anti-KAHRP (green) and anti-GFP (green). Nuclei were stained with DAPI (blue). Projections of Z stacks are shown. Scale bar = 5 μm. (B) Sheared remnant membranes from RBCs infected with 3D7 and GEXP07 were labeled with anti-KAHRP (red) and anti-GFP (green). CellMask (CM) reveals each of the membrane discs in the sample (gray). Scale bar = 5 μm. (C) Quantification of the mean fluorescence intensity (MFI) of anti-GFP signal on uninfected RBC (uRBC), infected RBC (iRBC), and the slide background (Bg). 3D7, *n* = 64 cells; GEXP07-GFP, *n* = 76 cells. Fluorescence values are in arbitrary units (a.u). (D) Western analysis of trypsin-cleaved 3D7-infected and GEXP07-GFP-infected RBCs. Saponin (SAP), Triton X-100 (Tx100), and SDS solubilized fractions were extracted from either PBS mock-treated or trypsin-treated samples. The membranes were cut to allow differential probing of proteins of different sizes. The regions of the membrane containing full-length GEXP07-GFP and the potential cleavage product were probed separately. Blue arrow = PfEMP1 cleavage product. Red arrow = full-length PfEMP1 and spectrin cross-reaction.

To determine or confirm the locations of a number of the identified proteins, we generated transfectants expressing GFP fusions of five uncharacterized or partially characterized proteins (GEXP07, GEXP10, PTP5, PTP6, and *PF*3D7_1353100) and two proteins that were previously reported to be Maurer’s cleft-located proteins (REX1 [17] and MAHRP1 [34]), each under the control of the *CRT* (chloroquine resistance transporter) promoter (Fig. 2). Probing of an immunoblot with anti-GFP confirmed the expression of chimeric proteins that migrate at close to the calculated molecular masses (Fig. S1C). Using live-cell microscopy, we visualized each of the GFP-tagged proteins as puncta in the host RBC cytoplasm, consistent with a Maurer’s cleft association (Fig. 2). Immunofluorescence microscopy of samples co-labeled with antibodies recognizing GFP and REX1 confirmed that the proteins were directed to the Maurer’s clefts (Fig. S2A). The fluorescence profile for GEXP07-GFP is quite different from that of the RBC membrane-associated protein, KAHRP (Fig. 3A). This is at odds with a previous study, which suggested that GEXP07 and GEXP10 are present at the RBC surface (29). In an effort to determine if a subpopulation of GEXP07-GFP is surface located, we employed a method for examining the cytoplasmic surface of infected RBC membranes which we have previously used to probe the RBC membrane-located population of GFP-tagged PfEMP1 (2). Infected 3D7 wild-type and GEXP07-GFP-infected RBCs were tightly linked to lectin-coated slides and subjected to shearing followed by probing with anti-KAHRP and anti-GFP antibodies. While a weak background signal was observed, the fluorescence signal from the RBC membranes of GEXP07-GFP parasites was not significantly different from that of 3D7 wild-type-infected RBCs (Fig. 3B and C).

10.1128/mBio.03320-19.2 FIG S2 Immunofluorescence microscopy and Western blotting of GFP-tagged proteins. (A) The GFP-tagged transfectant-infected RBC smears were fixed in acetone/methanol (9:1) at –20°C. Samples were labeled with anti-GFP (green) to localize the GFP-tagged protein and with anti-REX1 (red) to label the Maurer’s clefts. Parasite nuclei were stained with DAPI (blue). Scale bars = 5 μm. (B to E) Infected RBCs were solubilized in 1% Triton X-100 and subjected to SDS-PAGE. Input loading was 4% of the pellet loading. Green arrowheads indicate the full-length GFP-tagged proteins. Blue arrowheads indicate co-precipitated proteins. (B) 3D7 wild-type, REX1-GFP. (C) PTP6-GFP. (D) GEXP10-GFP, GEXP07-GFP, MAHRP1-GFP (M1), and *PF*3D7_1353100-GFP (*PF*13) IPs probed with anti-GFP antibody. (E) GEXP10-GFP, GEXP07-GFP, and MAHRP1-GFP (M1) IPs probed with anti-MAHRP1 antibodies. Input (In) and IP (IP) fractions are shown. Molecular masses are in kilodaltons. Download FIG S2, PDF file, 0.2 MB. Copyright © 2020 McHugh et al. 2020 McHugh et al. This content is distributed under the terms of the Creative Commons Attribution 4.0 International license.

We next performed a trypsin cleavage assay to detect GEXP07-GFP at the surface of the RBC. GEXP07-GFP-infected RBCs were treated with trypsin and then subjected to successive extractions in Triton X-100 and SDS. As reported previously, an antiserum against the acidic terminal segment (ATS) detects the surface-exposed population of PfEMP1 (Fig. 3D, top panel) (35). The proposed topology of GEXP07 at the RBC membrane exposes a small extracellular loop which contains a lysine residue that would be cleaved by trypsin if exposed. Cleavage of GEXP07 is expected to generate a 32.5-kDa GFP-containing fragment. In contrast, while full-length GEXP07-GFP was detectable in all fractions (Fig. 3D, second panel), no GEXP07 fragment was detected, even in overexposed blots of the expected region of the gel (Fig. 3D, third panel). These data argue against exposure of GEXP07-GFP at the RBC surface, at least in these trophozoite-stage parasites.

### Co-IP of Maurer’s cleft proteins reveals a compartmentalized network of protein interaction clusters.

**FIG 4:**
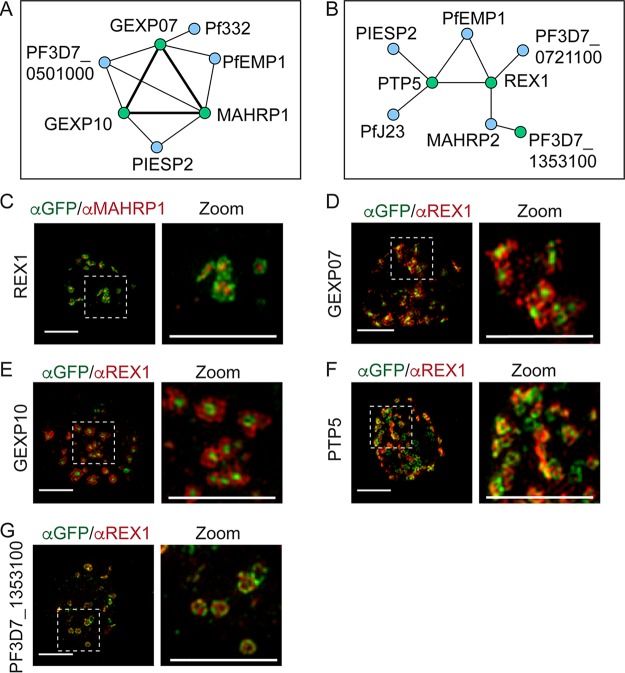
Maurer’s cleft proteins interact to form distinct protein clusters. (A and B) Protein interaction maps highlighting a putative *Pf*EMP1 loading hub comprising GEXP07, MAHRP1, and GEXP10 (A) and a putative unloading hub comprising REX1, PTP5, SEMP1, and *PF*3D7_1353100 (B). Green nodes represent the GFP-tagged proteins. Double-thickness edges indicate reciprocal co-precipitation (see Fig. S3 for full-network maps). (C) 3D-SIM analysis of REX1-GFP-infected RBCs fixed and labeled with anti-GFP (green) and anti-MAHRP1 (red). (D to G) 3D-SIM analysis of transfectant-infected RBCs expressing GFP-tagged GEXP07, GEXP10, PTP5, and *Pf*3D7_1353100 that were fixed and labeled with anti-GFP (green) and anti-REX (red) antibodies. Maximum projections of Z-stacks are displayed. Scale bars = 3 μm, zoom scale bar = 3 μm.

We performed co-IP experiments on our GFP-tagged transfectants. Western analysis confirmed the enrichment of the GFP-tagged bait proteins (Fig. S2B to D). In each of the lines analyzed, the bait protein and several interacting proteins were identified (see Table S2 to S8; see also Data Sets 2 to 8). The protein interaction networks were analyzed using Navigator (Fig. 4A and B; see also Fig. S3) (36). Combining the resulting data with data from other studies that performed IPs with epitope-tagged PTP1, SEMP1, SBP1, and *Pf*EMP1, a complex map of interactions was revealed (Fig. S3B) (15, 16, 31, 37). We identified 2 protein clusters that each contained Maurer’s cleft proteins that are known to have roles in *Pf*EMP1 trafficking and in connecting to uncharacterized proteins and to *Pf*EMP1 itself (Fig. 4A and B).

10.1128/mBio.03320-19.3 FIG S3 Complete network map of co-IP experiments. (A) Connectivity map of the GFP-tagged bait proteins and their interactions with proteins identified in the co-IP experiments. (B) Connectivity map combining IP data for SBP1, SEMP1, PTP1, and *Pf*EMP1 from the literature. Green nodes represent the GFP-tagged proteins. Blue nodes represent exported interacting proteins. Purple nodes represent proteins within the parasitophorous vacuole. Double-width lines represent reciprocal IP. Download FIG S3, PDF file, 0.1 MB. Copyright © 2020 McHugh et al. 2020 McHugh et al. This content is distributed under the terms of the Creative Commons Attribution 4.0 International license.

For example, previous work showed that MAHRP1 is needed for efficient loading of *Pf*EMP1 into the Maurer’s clefts (38). We found that MAHRP1-GFP co-precipitated GEXP07 and GEXP10, while GEXP07-GFP and GEXP10-GFP co-precipitated MAHRP1 (Table S2 to S4; see also Data Sets 2 to 4). These interactions were confirmed by Western blotting (Fig. S2E). GEXP10-GFP and MAHRP1-GFP also precipitated PIESP2, while GEXP07-GFP precipitated *Pf*322, consistent with its known Maurer’s cleft location. GEXP07-GFP and GEXP10-GFP also precipitated *Pf*EMP1 (Table S2 to S4; see also Data Sets 2 to 4), suggesting a potential role for these proteins in *Pf*EMP1 trafficking.

Three-dimensional structured illumination microscopy (3D-SIM) provides an 8-fold increase in volume resolution, which enhances the analysis of compartments (such as the Maurer’s clefts) that have dimensions close to the resolution limit of conventional microscopy. 3D-SIM revealed that REX1 was located around the perimeter of the clefts, while MAHRP1 exhibited a more central location (Fig. 4C; see also Fig. S4A), in agreement with our previous report (11). GEXP07-GFP and GEXP10-GFP are concentrated in the central region of the Maurer’s cleft surrounded by REX1 (Fig. 4D and E; see also Fig. S4B). The profile for antibodies recognizing the ATS region of *Pf*EMP1 exhibited partial overlap of that for GEXP10-GFP (Fig. S4C).

10.1128/mBio.03320-19.4 FIG S4 Whole-cell analyses of GFP-tagged transfectants by 3D-SIM. (A) REX1-GFP-infected RBCs were fixed and labeled with anti-GFP (green) and anti-MAHRP1 (red). (B) The remaining GFP-tagged transfectant-infected RBCs were fixed and labeled with anti-GFP (green) and anti-REX (red). Average projections of z-stacks are displayed. The merged image and zoom as displayed in Fig. 3 are shown. Merge scale bars = 3 μm. Zoom scale bars = 3 μm. (B) 3D-SIM imaging of GEXP10-GFP and REX1-GFP (red) parasites co-stained with ATS antibodies (*Pf*EMP1) (green). Merged images and a zoom of the merge are shown. Merge scale bars = 3 μm. Zoom scale bars = 1 μm. Download FIG S4, PDF file, 0.1 MB. Copyright © 2020 McHugh et al. 2020 McHugh et al. This content is distributed under the terms of the Creative Commons Attribution 4.0 International license.

The interaction analysis revealed a second protein cluster containing REX1-GFP, PTP5-GFP, and *PF*3D7_1353100 (Fig. 3B). REX1-GFP co-precipitated *Pf*EMP1 (Table S5; see also Data Set 5), consistent with the known role for REX1 in trafficking *Pf*EMP1 from the Maurer’s clefts to the RBC membrane (35, 39). A co-IP with PTP5-GFP co-precipitated REX1, PIESP2, and *Pf*J23 (Table S6; see also Data Set 6). These data point to the existence of a protein interaction network comprised of *Pf*EMP1, REX1, PTP5, and PIESP2, potentially functioning at the cleft periphery (Fig. 4B). REX1-GFP and *PF*3D7_1353100-GFP also co-precipitated the tether protein, MAHRP2 (Table S7; see also Data Set 7).

3D-SIM imaging of PTP5-GFP and *PF*3D7_1353100-GFP cell lines revealed a dotted pattern at the periphery of the Maurer’s cleft cisternae, partially overlapping or alternating with the REX1 signal (Fig. 4F and G; see also Fig. S4B). Similarly, *Pf*EMP1 (ATS labeling) partly overlapped REX1 and *Pf*EMP1 at the cleft periphery (Fig. S4C).

Components of the PVM translocation machinery were observed in IPs performed with GEXP07-GFP (HSP101, PTEX150), suggesting the occurrence of interactions with these proteins during the export processes. IPs performed with PTP6-GFP showed a number of interacting proteins, including MESA and STARP and three proteins of unknown function, *PF*3D7_0702500, *PF*3D7_1002000, and *PF*3D7_0301700. This protein interaction network showed no connectivity to the clusters described above (Table S8; see also Data Set 8).

### GEXP07 gene knockout results in altered trafficking of exported proteins and a growth advantage.

As we were interested in identifying proteins that may be involved in trafficking *Pf*EMP1, we selected the genes encoding GEXP07 and GEXP10 (which IP each other and *Pf*EMP1) as candidates for genetic disruption. These genes have been previously reported to be refractory to deletion methods using conventional approaches (21, 28). In this work, we used a CRISPR-Cas9 approach to disrupt the genes in CS2 parasites (Fig. S5). We were unable to obtain a knockout of GEXP10 in two separate attempts, supporting results from a recent genome-wide transposon screen which suggested that GEXP10 is essential (40). In contrast, a knockout was obtained for GEXP07, with disruption of the native locus confirmed by PCR (Fig. S5).

10.1128/mBio.03320-19.5 FIG S5 Generation and validation of the ΔGEXP07 parasite line. (A) Gene disruption and PCR validation schematic. GOI = gene of interest; HR1 = homology region 1; HR2 = homology region 2. Crossed lines indicate a crossover event. Arrows, primers; bracketed lines, expected amplicon size. (B) PCR amplicons. The letters refer to primers shown in panel B. KO = knockout. The first lane features the marker. Download FIG S5, PDF file, 0.2 MB. Copyright © 2020 McHugh et al. 2020 McHugh et al. This content is distributed under the terms of the Creative Commons Attribution 4.0 International license.

**FIG 5:**
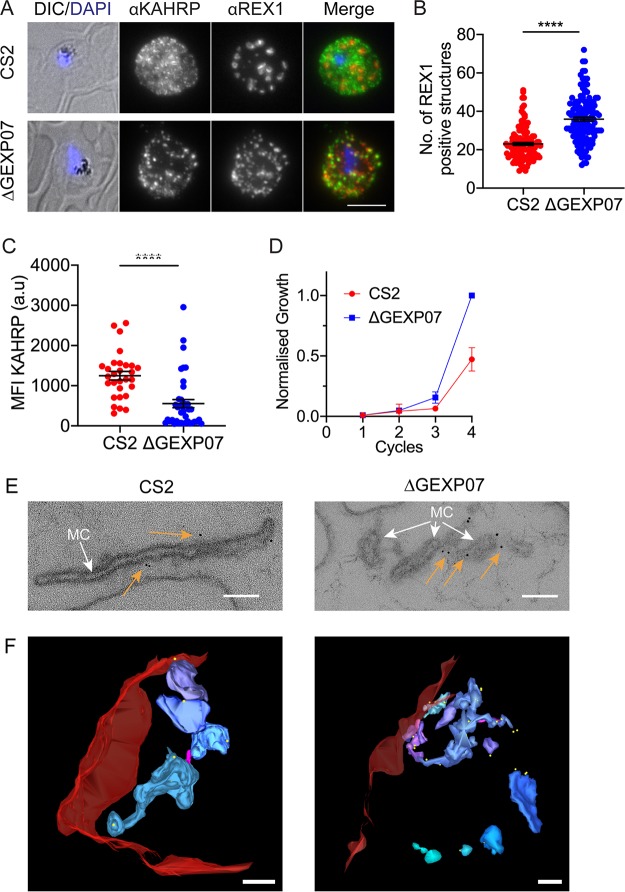
Knockout of GEXP07 alters parasite growth and Maurer’s cleft architecture. (A) Infected RBCs were fixed and probed with anti-KAHRP (green) and counter stained with anti-REX1 (red). Nuclei were stained with DAPI (blue). CS2 = parent line. Projections of Z stacks are shown. Scale bar = 5 μm. (B) Quantification of Maurer’s cleft number. The data plotted represent means ± standard errors of the means of results from 3 separate biological experiments. A total of 121 CS2 and 132 GEXP07 cells were quantified (unpaired *t* test; **** = *P* < 0.0001). (C) Quantification of the mean fluorescence intensity (MFI) of KAHRP fluorescence (unpaired *t* test; ****, *P* < 0.0001). CS2, *n* = 29 cells. ΔGEXP07, *n* = 41 cells. Fluorescence values are in arbitrary units (a.u). (D) Parasite growth assay measuring proliferation over 4 asexual cycles. Growth was assessed by staining infected RBCs with SYTO-61 and subsequent flow cytometry analysis. The data have been normalized and are expressed relative to the ΔGEXP07 parasite line (*n* = 4 experiments performed in triplicate). (E) EqtII-permeabilized wild-type CS2 and GEXP07 knockout transfectants were labeled with antibodies recognizing REX1 followed by immunogold labeling and were prepared for electron microscopy. Images have been cropped around Maurer’s clefts (white arrows). Additional images displaying the Maurer’s cleft morphologies are shown in Fig. S6A. Gold arrows point to gold particles. Scale bar 100 nm. (F) Rendered 3D models of Maurer’s clefts generated from electron tomograms. Red = RBC; magenta stalk = tether; pastel hues = independent clefts. Scale bar = 200 nm. Translations through the virtual sections of the tomograms and rotations of the rendered models can be seen in Movies S2 and S3.

We examined ΔGEXP07-infected RBCs by immunofluorescence microscopy, using antibodies to REX1 and KAHRP (Fig. 5A). An increase in the number of REX1-positive puncta was observed (Fig. 5B) suggesting a change in Maurer’s cleft morphology. The labeling pattern for KAHRP also appeared more punctate (Fig. 5A), and quantification revealed a significant decrease in the overall KAHRP fluorescence intensity of ΔGEXP07-infected RBCs compared to the wild-type CS2 (Fig. 5C). We next analyzed the growth of the ΔGEXP07 parasite line compared with the CS2 parent parasites. The ΔGEXP07 parasite line grew faster than the parent line, with a more than 2-fold difference observed after four parasite cycles (Fig. 5D).

10.1128/mBio.03320-19.6 FIG S6 Transmission electron microscopy of CS2-infected and ΔGEXP07-infected RBCs. Additional examples of the Maurer’s cleft morphologies for wild-type CS2 (A) and ΔGEXP07 (B) parasites. TEM of equinatoxin-treated RBCs was performed with results highlighting the fragmented Maurer’s clefts. MC = Maurer’s clefts. Scale bars, 500 nm. Download FIG S6, PDF file, 2.6 MB. Copyright © 2020 McHugh et al. 2020 McHugh et al. This content is distributed under the terms of the Creative Commons Attribution 4.0 International license.

10.1128/mBio.03320-19.9 MOVIE S2 CS2 electron tomography translation and rendered model. (Part 1) Movie translating through a reconstructed z-stack for CS2-infected red blood cells that had been subjected to equinatoxin II permeabilization and probed with anti-REX1 (1:50) followed by Aurion protein A (EM-grade 6-nm-diameter gold JA806-111 particles; 1:15). (Part 2) A 360° rotation around the *y* axis of CS2 Maurer’s cleft 3D structure reconstructed and rendered in IMOD. Red = RBC; fuchsia stalk = tether; pastel hues = independent clefts; scale bar = 200 nm. Download Movie S2, AVI file, 14.2 MB. Copyright © 2020 McHugh et al. 2020 McHugh et al. This content is distributed under the terms of the Creative Commons Attribution 4.0 International license.

10.1128/mBio.03320-19.10 MOVIE S3 Translation through an electron tomogram and rendered model of ΔGEXP07 transfectants. (Part 1) Movie translating through a reconstructed z-stack for ΔGEXP07-infected red blood cells that had been subjected to equinatoxin II permeabilization and probed with anti-REX1 (1:50) followed by Aurion protein A (EM-grade 6-nm-gold JA806-111 particles; 1:15). (Part 2) A 360° rotation around the *y* axis of ΔGEXP07 Maurer’s cleft 3D structure reconstructed and rendered in IMOD. Red = RBC; fuchsia stalk = tether; pastel hues = independent clefts; scale bar = 100 nm. Download Movie S3, AVI file, 15.8 MB. Copyright © 2020 McHugh et al. 2020 McHugh et al. This content is distributed under the terms of the Creative Commons Attribution 4.0 International license.

### Knockout of GEXP07 results in altered Maurer’s cleft morphology.

RBCs infected with the ΔGEXP07 parasite line were lightly fixed and permeabilized with the pore-forming toxin equinatoxin II (EqtII) (41, 42) to release hemoglobin and to permit the introduction of primary antibodies recognizing REX1.

Transmission electron microscopy (TEM) was performed on thin sections prepared from CS2-infected and ΔGEXP07-infected RBCs. The Maurer’s clefts are observed as single slender cisternae with an electron-lucent lumen and an electron-dense coat in wild-type CS2 (Fig. 5C; see also Fig. S6A). In ΔGEXP07-infected RBCs, distorted and fragmented structures were observed (Fig. 5C; see also Fig. S6A). These fragments labeled with immunogold-labeled anti-REX1 antibodies, confirming a Maurer’s cleft origin (gold arrows; Fig. 5C). The 3D architecture of the Maurer’s cleft fragments was examined using electron tomography (Fig. 5D). The rendered images reveal the fragmented nature of the Maurer’s clefts compared to wild-type CS2. Each separate cleft structure is rendered in a different pastel hue; the RBC (red) and the tethers (magenta) are also displayed (Fig. 5D). The morphological changes are best appreciated in translations through the virtual sections of the tomogram and rotations of the rendered models (see Movies S2 and S3).

### ΔGEXP07 parasite-infected RBCs have altered knob morphology.

**FIG 6:**
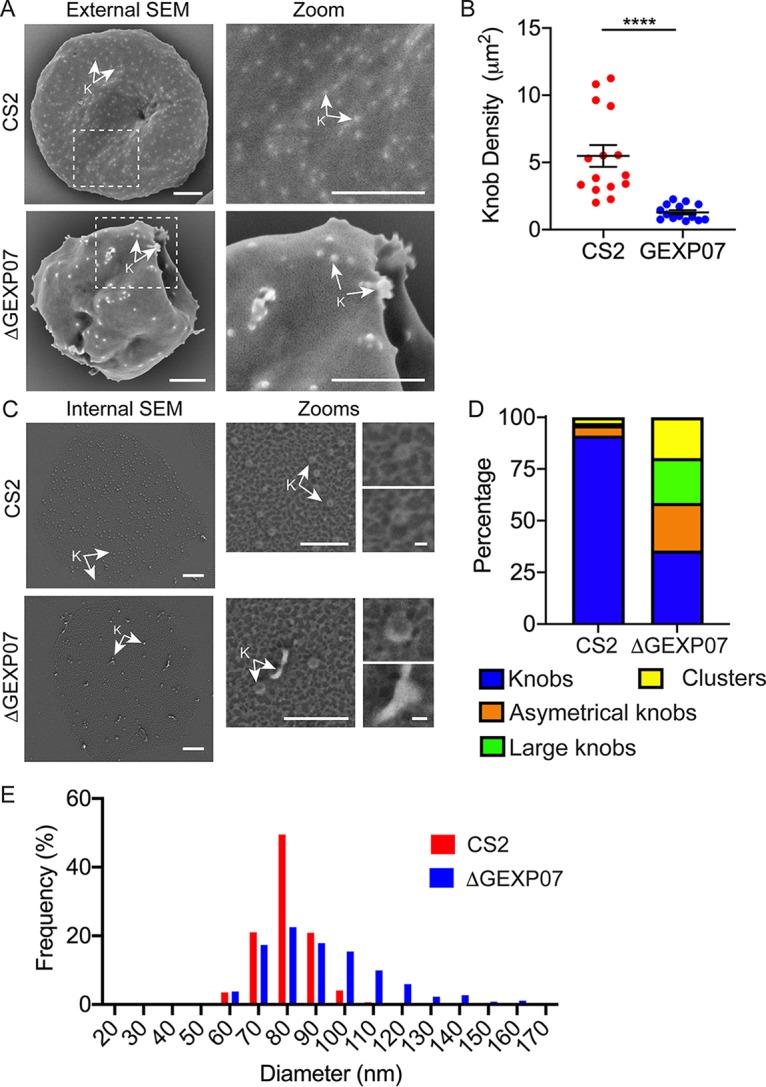
Examination of ΔGEXP07-infected RBCs reveals altered knob morphology. (A) Mid-trophozoite-stage infected RBCs were fixed in 2.5% glutaraldehyde–PBS and prepared for SEM of the exterior surface. Scale bar = 1 μm. (B) Quantification of the number of knob-like structures observed in the external SEM. Data represent the mean number of structures per μm^2^ (*n* = 15 cells per sample, minimum 3 areas per cell) (unpaired *t* test; ****, *P* < 0.0001). (C) Late-trophozoite-stage-infected RBCs were immobilized onto glass slides. Shearing under hypotonic conditions left remnant membrane discs that were fixed, dehydrated, coated with gold, and imaged using SEM. Knobs (K) are indicated with arrows. Scale bar = 1 μm; zoom 1 scale bar = 500 nm; zoom 2 scale bar = 50 nm. (D) Quantification of the knob morphologies observed by internal SEM imaging. These are defined as normal knobs (blue), asymmetrical knobs (orange), large knobs (green), and knob clusters (yellow). Example images of these morphologies are shown in Fig. S7B. (E) Graph showing the frequency distribution of knob sizes from wild-type CS2- and ΔGEXP07-infected RBCs. A bin size of 10 nm was used; *n* = 760 (ΔGEXP07) or 1,098 (CS2).

Scanning electron microscopy (SEM) of intact ΔGEXP07-infected RBCs revealed enlarged knobs with a highly aberrant morphology, as well as knob clusters (Fig. 6A). RBCs infected with ΔGEXP07 parasites had fewer knob structures (1.3 ± 0.2 per μm^2^) than were seen with wild-type CS2 (5.5 ± 1 per μm^2^) (Fig. 6B). Thin-section TEM confirmed the altered knob morphology (Fig. S7A).

10.1128/mBio.03320-19.7 FIG S7 Thin-section transmission electron microscopy of infected RBC morphology and scanning EM of knob morphology. (A) Thin-section TEM highlighting the knob and MC morphologies. Parasite features are labeled. K = knobs; MC = Maurer’s clefts. Scale bars, 500 nm. (B) Scanning electron microscopy images of normal, large, clustered, and asymmetrical knobs. Scale bars = 100 nm. Download FIG S7, PDF file, 0.5 MB. Copyright © 2020 McHugh et al. 2020 McHugh et al. This content is distributed under the terms of the Creative Commons Attribution 4.0 International license.

To investigate the architecture of the knobs in more detail, we made use of a recently developed method for imaging knobs at the cytoplasmic surface of infected RBCs (2). Remnant discs of sheared infected RBCs were fixed, dehydrated, coated with gold, and then imaged using high-resolution SEM. The membrane protein network, containing spectrin and actin, was evident as bright skeletal elements over dark patches of background membrane (Fig. 6C). The knobs appear as raised, dimpled disk-shaped structures that are closely integrated into the skeletal meshwork (Fig. 6C, arrows). About 75% of the knobs were malformed in the ΔGEXP07-infected RBCs, presenting as asymmetrical, enlarged, and clustered structures (Fig. 6D; see also Fig. S7B). The knobs in the ΔGEXP07-infected RBC membranes exhibited a larger average diameter (90 ± 1 nm) than the CS2 wild-type parent (80 ± 1 nm) and a broader size distribution (Fig. 6E).

**FIG 7:**
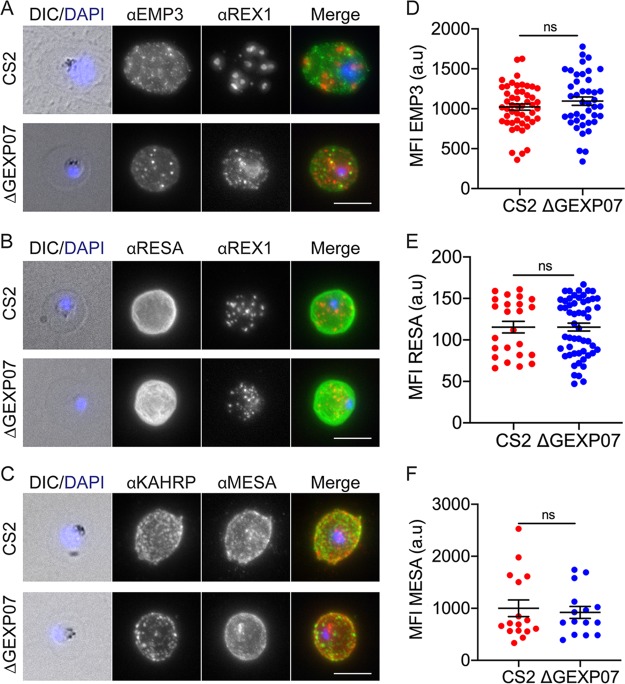
GEXP07 knockout has no effect on the location of other RBC-locating proteins. Immunofluorescence assays were performed using antibodies to PfEMP3, RESA, and MESA. (A and B) anti-PfEMP3 (green) and anti-RESA (green) counter stained with anti-REX1 (red). (C) Anti-MESA (red) counter stained with anti-KAHRP (green). Nuclei were stained with DAPI (blue). Projections of Z stacks are shown. Scale bar = 5 μm. (D to F) Quantification of the mean fluorescence intensity (MFI) of PfEMP3, RESA, and MESA (unpaired *t* test; = ns, not significant). (D) PfEMP3. CS2 *n* = 50 cells and ΔGEXP07 *n* = 42 cells. (E) RESA. CS2 *n* = 24 cells and ΔGEXP07 *n* = 54 cells. (F) MESA. CS2 *n* = 16 cells and ΔGEXP07 *n* = 15 cells. Fluorescence values are in arbitrary units (a.u).

To confirm that the observed aberrant behavior of KAHRP was not due to a global decrease in the level of protein export, immunofluorescence microscopy was performed using antibodies to RESA, MESA, and PfEMP3, all of which located at the RBC membrane (Fig. 7A to C). Quantification of these images showed no significant change in their mean fluorescence intensity in the absence of GEXP07 (Fig. 7D to F).

### Knockout of GEXP07 reduces *Pf*EMP1 surface expression and binding to chondroitin sulfate-A.

**FIG 8:**
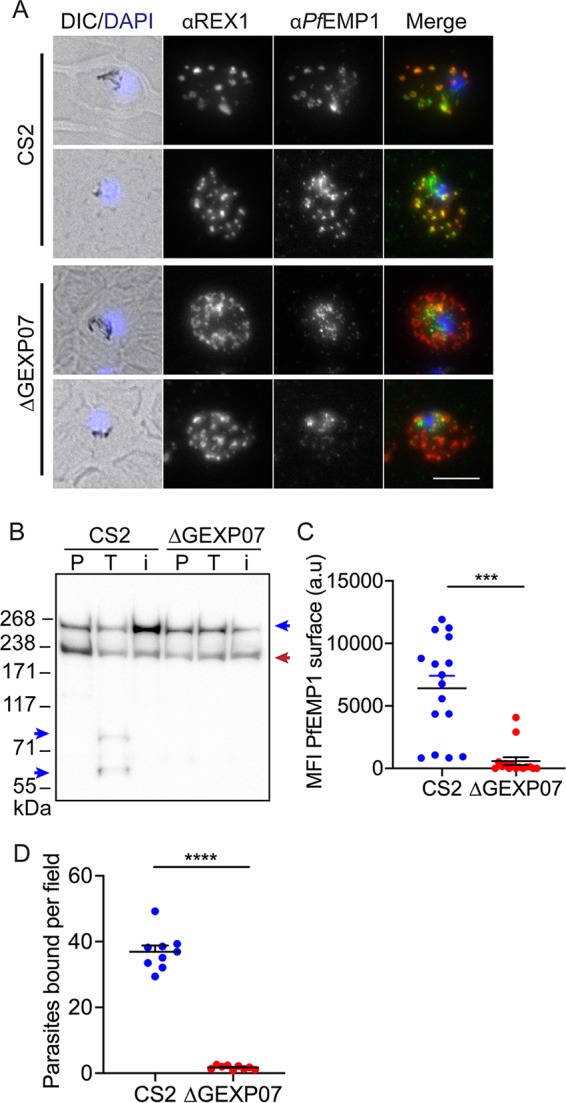
Genetic knockout of GEXP07 led to a loss of *Pf*EMP1 at the RBC surface and impaired adhesion. (A) Wild-type CS2 and GEXP07 knockout-infected RBCs were fixed and probed with anti-*Pf*EMP1 (green) and counter stained with anti-REX1 (red). Nuclei were stained with DAPI (blue). CS2 = parent line. Projections of Z stacks are shown. Scale bar = 5 μm. (B) Whole infected RBCs were treated with trypsin (T) or with trypsin plus trypsin inhibitor (i) and solubilized in Triton X-100, and the resultant pellet representing RBC membrane-embedded *Pf*EMP1 was subjected to Western blotting and probed with anti-ATS. Full-length *Pf*EMP1 (350 kDa) and ATS bands are indicated with blue arrows. Bands at ∼240 kDa represent cross-reaction with spectrin (red arrow). An ∼70-kDa cross-reactive species can be seen in all samples. (C) Flow cytometry analysis of infected RBCs labeled with antibodies to the external domains of *Pf*EMP1. Experiments were performed at least in triplicate on 3 separate occasions. Errors bars represent standard errors of the means (unpaired *t* test, *P* = 0.0002). The mean fluorescence intensity (MFI) values are plotted. (D) Mid-trophozoite-stage-infected RBCs were examined for their ability to bind to chondroitin sulfate-A under physiological flow conditions. The average number of adherent infected RBCs in 10 fields of view was assessed from triplicate experiments performed on 3 separate occasions. The data from each repeat are plotted. Unpaired *t* test; **** = *P* < 0.0001. Error bars represent standard errors of the means.

To determine if *Pf*EMP1 trafficking was affected in the ΔGEXP07-infected RBCs, we performed immunofluorescence microscopy using antibodies directed toward the conserved ATS of *Pf*EMP1. Wild-type CS2-infected RBCs exhibited the characteristic localization of *Pf*EMP1 at the Maurer’s clefts, while *Pf*EMP1 labeling was much weaker in the ΔGEXP07-infected RBCs (Fig. 8A).

We next examined if *Pf*EMP1 is displayed at the surface of ΔGEXP07-infected RBCs by the use of a trypsin cleavage assay (8, 32). Whole infected RBCs were mock treated with phosphate-buffered saline (PBS) (P) or treated with trypsin (T) or with trypsin plus inhibitor (i) followed by Triton X-100 solubilization. In the PBS-treated portion (P) and the protease-inhibited CS2 control (i), RBC membrane-embedded full-length *Pf*EMP1 was evident, with an apparent molecular mass of ∼270 kDa (Fig. 8B, top blue arrow). In the trypsin-treated CS2 sample (T), the surface-exposed pool of *Pf*EMP1 was depleted and two cleavage products of ∼75 kDa and ∼60 kDa (blue arrows) which correspond to the membrane-embedded conserved ATS region of *Pf*EMP1 were observed. These cleavage products were absent in the ΔGEXP07-infected RBC samples, which is consistent with loss of the surface-exposed pool of *Pf*EMP1 (Fig. 8B). A cross-reaction of the *Pf*EMP1 antibody with RBC spectrin at ∼220 kDa was observed (Fig. 8B, red arrow) (21).

To further examine surface presentation of *Pf*EMP1, we used a rabbit polyclonal serum that recognizes VAR2CSA on the surface of CS2 parasite-infected RBCs (43). A strong signal was observed in CS2 parasites and was reduced by 90% in the ΔGEXP07-infected RBCs (Fig. 8C).

The CS2 parent line expresses a fixed *Pf*EMP1 variant, VAR2CSA, which binds to chondroitin sulfate-A. We investigated the ability of infected RBCs to bind to immobilized chondroitin sulfate-A under physiologically relevant flow conditions. RBCs infected with ΔGEXP07 parasites showed a 95% lower level of adhesion than those infected with the CS2 parent (Fig. 8D). This provides further evidence that parasites are unable to traffic *Pf*EMP1 to the RBC surface in the absence of GEXP07.

**FIG 9:**
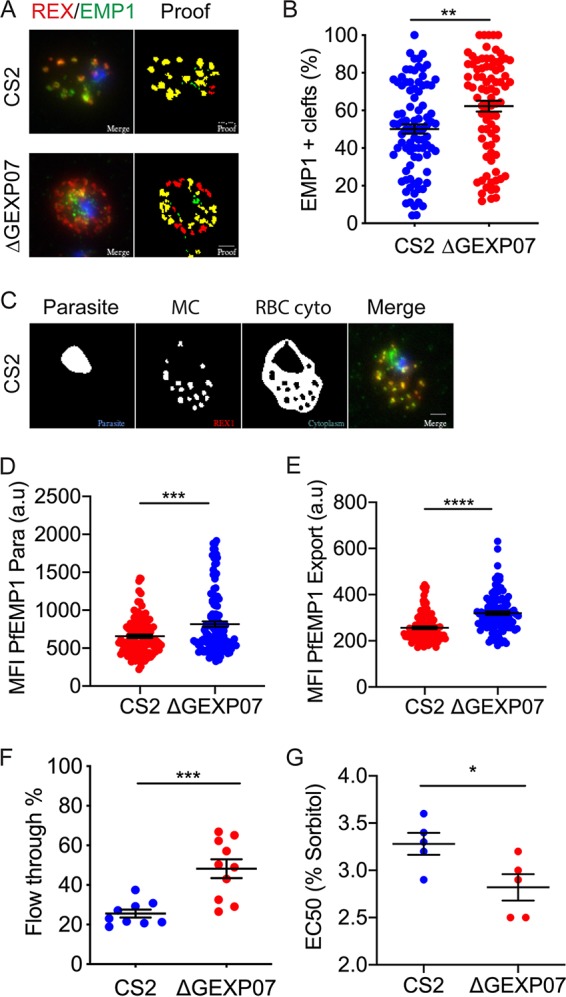
Gene knockout of GEXP07 led to a PfEMP1 trafficking defect, increased deformability, and increased cellular fragility. (A) Representative images illustrating the output from a custom macro used to quantify the percentage of Maurer’s clefts that are positive for PfEMP1. The original image is shown on the left and in Fig. 8A and the rendered macro output proof on the right. More examples and a full explanation of the macro are shown in the supplemental methods. (B) Quantitative analysis of the percentage of REX1 (red)-stained Maurer’s clefts that costained with PfEMP1 (green). The data shown represent percent overlap. CS2, *n* = 89 cells. ΔGEXP07, *n* = 82 cells. Unpaired *t* test; **, *P* = 0.0017. Error bars represent standard errors of the means. (C) Images highlighting the quantification strategy. The original image is shown on the left, and the masked areas for the parasite and the RBC cytoplasm (RBC cyto) are shown in the two center panels. The Maurer’s cleft (MC) mask was subtracted from the cytoplasm in order to measure PfEMP1 fluorescence present elsewhere than at the Maurer’s clefts. The mean fluorescence intensity (MFI) was measured for the parasite and RBC cyto areas. (D) The mean fluorescence intensity (MFI) of PfEMP1 within the parasite (Para). CS2, *n* = 113 cells. ΔGEXP07, *n* = 123 cells. Unpaired *t* test; **** = *P* < 0.0001. Error bars represent standard errors of the means. (E) The mean fluorescence intensity (MFI) of PfEMP1 within the RBC cytoplasm. CS2, *n* = 102 cells. ΔGEXP07, *n* = 105 cells. Unpaired *t* test; **** = *P* < 0.0001. Error bars represent standard errors of the means. (F) Spleen mimic filtration showing a significant increasing in filterability for the ΔGEXP07-infected RBCs. Experiments were performed in triplicate on 3 separate occasions. Unpaired *t* test, *P* = 0.0006. Error bars represent standard errors of the means. (F) Analysis of susceptibility to lysis in sorbitol solutions. Mid-trophozoite-stage-infected red blood cells were incubated in 1 of 12 solutions that included between 0% (wt/vol) and 5.5% (wt/vol) d-sorbitol, and the resulting level of parasitemia was measured by flow cytometry. Data representing EC_50_ values from triplicate experiments performed on 5 separate occasions are shown. The long horizontal line between the shorter horizontal lines represents the median.

Given the inability of the ΔGEXP07 parasites to traffic PfEMP1 to the RBC membrane, we next performed image quantification to determine where in the export pathway PfEMP1 was accumulating. We first assessed how many of the REX1-labeled cleft structures were also positive for PfEMP1. We saw a small but significant increase in the number of cleft structures co-labeled with PfEMP1 (Fig. 9B), suggesting an accumulation of PfEMP1 at the Maurer’s clefts in the ΔGEXP07 parasites. We also analyzed the intensity of fluorescence labeling of PfEMP1 within the parasite (Fig. 9C) and within the host RBC cytoplasm (Fig. 9D). These analyses showed significant increases in the amount of PfEMP1 labeling within both the parasite and the host cell cytoplasm compared to wild-type parasites (Fig. 9C and D).

### RBCs infected with ΔGEXP07 parasites have increased deformability and fragility.

We examined the ability of CS2- and ΔGEXP07-infected RBCs to filter through a bed of beads designed to mimic splenic fenestrations. RBCs infected with ΔGEXP07 parasites synchronized to 20 to 24 h post invasion (the window during which knob assembly and host cell remodeling occurs [2]) exhibited a 23% increase in filterability compared to the CS2 controls (Fig. 9E).

We next examined the sensitivity of infected RBCs to osmotic lysis. Mid-trophozoite-stage-infected RBCs were incubated in d-sorbitol solutions (0% to 5.5% [wt/vol]), and the remaining parasitemia was determined by flow cytometry. The 50% effective concentration (EC_50_) values were calculated for each of the experiments. We found that ΔGEXP07-infected RBCs are more sensitive to sorbitol-induced swelling and lysis than RBCs infected with wild-type CS2 (Fig. 9F).

## DISCUSSION

The suggestion that Maurer’s clefts play a role in transporting virulence proteins from the parasite to the RBC membrane was first put forward nearly 40 years ago (44); however, our understanding of the repertoire of cleft-associated proteins and their functions remains incomplete. A previous study examined the proteins present in the ghost fraction of trophozoite stage-infected RBCs and characterized two novel Maurer’s cleft proteins (14). More recently, proteomics approaches have been used to define the “exportomes” of both P. yoelii and P. berghei (45, 46). In this study, we built on this work and defined the composition of Maurer’s cleft in P. falciparum during the late ring stage of infection (14 to 18 h post invasion). During that time window, *Pf*EMP1 is trafficked to Maurer’s clefts and a subpopulation is transferred to the RBC membrane (8). A technical advantage of this time window is that the Maurer’s clefts are still mobile in the RBC cytoplasm, allowing separation from host and other parasite components.

Making use of a previously generated REX1-GFP-tagged line (17), we used GFP-Trap to affinity purify clefts released by hypotonic lysis. Mass spectrometry analysis identified enriched peptides from 18 proteins. Of these, 11 were previously shown to be Maurer’s cleft-located proteins, and 7 were potentially novel residents.

We also observed enrichment of host cell proteins in the Maurer’s clefts. The most highly enriched protein was annexin A4, which is involved in vesicle aggregation and the formation of lipid rafts (47). Other enriched human proteins include annexin A11, which binds to the calcium sensor calcyclin (33, 48), and copine-3 (a calcium-dependent phospholipid-binding protein), as well as VPS28, TSG101 (a component of the ESCRT-I complex), syntaxin-7 (a SNARE component), and VAC14 (a scaffold protein involved in phosphatidylinositol 3,5-bisphosphate synthesis). All of these proteins are involved in membrane trafficking. It is possible that these proteins play roles in Maurer’s cleft sculpting, vesicle formation, or *Pf*EMP1 trafficking, but their accumulation at the Maurer’s clefts remains to be verified.

We generated transfectants expressing GFP fusions of five putative Maurer’s cleft proteins and two known resident proteins. We confirmed that all of the tagged proteins were located at the Maurer’s clefts. The cell lines were then employed in an investigation of protein-protein interactions using GFP-Trap IP and protein identification by mass spectrometry. We identified hubs where newly identified or poorly characterized Maurer’s cleft proteins participate in networks with known *Pf*EMP1 trafficking proteins and, importantly, with *Pf*EMP1 itself.

For example, we identified a network involving *Pf*EMP1, REX1, PTP5, and the tether protein MAHRP2. This is consistent with previous evidence indicating that PTP5 co-precipitates with GFP-tagged *Pf*EMP1 and that REX1 and PTP5 are needed for *Pf*EMP1 trafficking to the RBC surface (21, 31, 35). We confirmed the interaction between REX1 and *Pf*EMP1, consistent with a previous study showing that GFP-tagged *Pf*EMP1 co-precipitates REX1 and vice versa (31). Our previous work showed that REX1 plays a role in trafficking *Pf*EMP1 from the clefts to the RBC membrane. Using 3D-SIM microscopy, we previously showed that REX1 is located at the edges of the Maurer’s clefts (11, 35, 39). In this work, we showed that PTP5 also locates at the cleft periphery, partially overlapping or intercalated with REX1 and *Pf*EMP1. Collectively, these data point to the presence of a protein hub that plays a role in the trafficking of *Pf*EMP1 from the Maurer’s clefts to the RBC membrane. Interestingly two members of this peripherally located hub, REX1 and *PF*3D7_1353100, co-precipitate MAHRP2. MAHRP2 is located on parasite-derived structures (called tethers) that have been proposed to link Maurer’s clefts to the RBC membrane (13).

A second cluster comprises MAHRP1, GEXP07, GEXP10, and PIESP2. 3D-SIM microscopy revealed that MAHRP1, GEXP07, and GEXP10 have overlapping physical locations in the central region of the Maurer’s clefts. Previous work has shown that MAHRP1 disruption prevents trafficking of *Pf*EMP1 to the Maurer’s clefts (38), suggesting a possible role for this protein hub in loading *Pf*EMP1 into the Maurer’s clefts.

We further characterized two proteins from this cluster, GEXP07 and GEXP10. These proteins show sequence similarity (32% identity) and are the only members of the Hyp8 gene family of exported *Plasmodium* proteins (49). They both contain a signal peptide and a PEXEL motif and have no orthologues in other *Plasmodium* species. The two proteins were initially reported to be exported to the RBC cytosol in gametocytes (27), but another report identified GEXP07 as a Maurer’s cleft-associated protein in asexual stages (28). Yet another antibody-based study reported that GEXP07 and GEXP10 are located at the RBC surface in the asexual blood stage (29) and are implicated in binding of infected RBCs to the chemokine CX3CL1. That study referred to them as CX3CL1-binding protein 2 (CBP2) and CBP1, respectively. In our work, we showed that both GEXP07 and GEXP10 are present at the Maurer’s cleft. We were unable to detect a RBC membrane-associated population of GEXP07-GFP. It remains possible that a small amount of GEXP07 is present at the RBC membrane but that the amount is below the limit of detection in our analyses.

Previous attempts to genetically disrupt GEXP07 and GEXP10 were unsuccessful, suggesting essentiality (21, 28). Using CRISPR-Cas9 and homology-directed repair, we were able to successfully generate a ΔGEXP07 parasite line but were unable to generate a GEXP10 knockout. The inability to knock out GEXP10 is consistent with data from a recent saturation-level mutagenesis study (40) which showed that GEXP10 is indispensable, based on the lack of *piggyBac* transposon insertion sites (mean insertion score of 0.26).

The ΔGEXP07 parasite line exhibited significant ultrastructural changes, with cleft fragmentation and swelling. GEXP07 has two predicted transmembrane domains, as with the previously characterized 2TM-MC (2TM-Maurer’s cleft) protein family. The 2TM-MC proteins have been reported to be oriented with the N- and C-terminal regions facing the RBC cytoplasm (50). If GEXP07 adopts an equivalent orientation, its 26-amino-acid loop (between the two transmembrane segments) would face the Maurer’s cleft lumen. It is possible that this loop sequence is involved in interactions that stabilize the Maurer’s clefts and that bring the two lamellae close together. The fragmentation of the Maurer’s clefts in the absence of GEXP07 is similar to the morphology observed previously when PTP1 was disrupted (16). The centrally located GEXP07 protein has an isoelectric point of 8.98, which is similar to that of PTP1 (8.79), while both of the peripherally located proteins, PTP5 and REX1, have an isoelectric point of 5.34. It is plausible that a loss of GEXP07 (or PTP1) from the central region of the cleft disrupts the electrostatic balance, leading to cleft destabilization and membrane vesiculation.

Knockout of GEXP07 also leads to the formation of enlarged aberrant knobs and knob clusters, which are sometimes located at the ends of membrane protrusions. Similarly, SEM imaging of the cytoplasmic face of infected RBC membranes revealed doughnut-shaped knob structures that were larger in ΔGEXP07-infected RBCs than in the CS2 parent.

The aberrant knob and Maurer’s cleft morphology is associated with a defect in the delivery of *Pf*EMP1 to the RBC membrane, as confirmed by trypsin cleavage assays and flow cytometry using antibodies to the ectodomain of *Pf*EMP1. As a consequence, the binding of the infected RBCs to chondroitin sulfate-A was reduced by 95%.

It is interesting to consider how the loss of a Maurer’s cleft-resident protein could result in aberrant knob morphology. While an earlier study using a chimeric KAHRP-GFP fusion (with a non-natural signal sequence) suggested that KAHRP might be trafficked to the knobs via the Maurer’s clefts (51), more recent studies have not supported this model. Thus, it seems unlikely that Maurer’s cleft-located GEXP07 plays a direct role in KAHRP trafficking. We considered the possibility that a small population of GEXP07 located at the RBC membrane, as previously proposed (29), could participate in knob assembly. However, we were unable to detect GFP-tagged GEXP07 at the RBC membrane.

An alternative possibility is that GEXP07 is required for the trafficking of a third-party protein required for knob assembly. A recent ultrastructural study visualized the knob complex as a spiral structure connected by multiple links to the RBC membrane skeleton and coated by an electron-dense layer that likely represents KAHRP (52). An earlier cryo-electron tomography study revealed branched actin filaments (each ∼500 nm in length) that connected the Maurer’s clefts to the RBC membrane skeleton in the region of the knobs (12). Recent work in our laboratory suggested that knobs are generated by the trafficking of individual KAHRP-containing modules to the RBC membrane skeleton followed by assembly of KAHRP modules into ring-shaped complexes that sit at the base of the physical knob structure (2). The composition of the spiral core remains unknown. One possibility is that correctly formed Maurer’s clefts may be needed for delivery of a knob assembly factor.

The ΔGEXP07 parasite line exhibited a growth advantage compared to wild-type CS2 parasites, perhaps consistent with the general observation that knob-negative parasites outgrow knob-positive parasites in culture. The abnormal knob morphology was found to be associated with increased deformability, suggesting that the reduced number of these aberrant knobs resulted in an inability to contribute the strain-hardening effects associated with wild-type knobs (53). Of interest, we found that ΔGEXP07-infected RBCs were more sensitive to osmotic changes, suggesting that the abnormal knobs weaken the membrane structure.

The current model for *Pf*EMP1 trafficking suggests that it is trafficked across the RBC cytoplasm as a chaperoned complex and then loaded into the membrane at the Maurer’s clefts (54). Forward trafficking delivers *Pf*EMP1 into the RBC membrane bilayer, from which it moves laterally to the knobs to form the mature virulence complex (2). The exact mechanism of *Pf*EMP1 unloading and trafficking is not known, but it has been speculated that *Pf*EMP1-loaded vesicles bud from the Maurer’s clefts and traffic along remodeled actin filaments or tethers. In the ΔGEXP07-infected RBCs, we observed a dramatic fragmentation of the Maurer’s clefts. We propose that this compromises the trafficking of both PfEMP1 and a knob assembly factor. GEXP07 is a basic protein located in the central region of the Maurer’s clefts. It is possible that GEXP07 interacts with the acidic C-terminal segment of *Pf*EMP1 and facilitates loading into the Maurer’s cleft membrane. It may then transfer *Pf*EMP1 to REX1 (at the Maurer’s cleft periphery) for onward delivery. Further studies are required to fully elucidate the role of GEXP07 in virulence protein trafficking.

In summary, we have developed a novel method for enriching Maurer’s clefts from late-ring-stage P. falciparum-infected RBCs. We identified a number of novel Maurer’s cleft proteins, expanding the repertoire of resident proteins at these structures. We characterized different protein hubs that may function in the trafficking of *Pf*EMP1 and determined their spatial organization. In particular, we identified interacting proteins located in the central region of the Maurer’s clefts that may be involved in loading *Pf*EMP1 into the Maurer’s cleft membrane. In the absence of one of these proteins, GEXP07, the Maurer’s clefts swell and fragment, the knobs enlarge and clump, and *Pf*EMP1 trafficking to the RBC membrane fails. RBCs infected with GEXP07-disrupted parasites are more deformable and more sensitive to sorbitol lysis, and the parasites possess a growth advantage. A more detailed understanding of the steps in the export pathway may reveal new strategies to target the parasite by blocking the delivery of *Pf*EMP1 to the infected RBC surface.

## MATERIALS AND METHODS

All of the tables are available for download from figshare using the following link: https://doi.org/10.26188/5e4f42d4c65fe. All data sets are available for download from figshare using the following link: https://doi.org/10.26188/5e4f43123b214. Additional supplemental descriptions of the methods used, including detailed explanations of the fluorescence quantification and the macros, can be downloaded from figshare using the following links: https://doi.org/10.26188/5e4f4271c4c31 and https://doi.org/10.26188/5e4f41364567f.

### Plasmodium falciparum culture.

Parasites were cultured as previously described (55). Briefly, P. falciparum cell lines were cultured in 5% human O^+^ red blood cells (Australian Red Cross Blood Service) in RPMI medium-GlutaMAX-HEPES (Invitrogen) supplemented with 5% (vol/vol) human serum (Australian Red Cross Blood Service), 0.25% (wt/vol) AlbuMAX II (Invitrogen), 200 μM hypoxanthine, 10 mM d-glucose (Sigma), and 20 μg/ml gentamicin (Sigma). Mature-stage parasites were enriched from parasite culture using Percoll purification or magnetic separation (56, 57). Ring-stage parasites were enriched by treatment with 5% d-sorbitol (58). Knob-positive parasites were enriched by flotation on Gelofusine (Braun) as previously described for gelatin (59). Transfectants containing the pGLUX plasmids were maintained in the presence of 5 nM WR99210 (Jacobus Pharmaceuticals). The ΔGEXP07 parasite line was maintained on 2 nM DSM1 (BEI Resources).

### Plasmid construction and transfection.

To create the GFP-tagged transfectant parasites used in this study, the full-length genes (minus the stop codon) were amplified from 3D7 genomic DNA (gDNA) using primers incorporating XhoI and KpnI restriction enzyme cleavage sites at the 5′ and 3′ ends of the gene (see Table S9 at https://doi.org/10.26188/5e4f43123b214). Sequence-verified DNA was then directionally cloned into the XhoI and KpnI sites of the pGLUX vector.

To generate the GEXP07 knockout cell lines, two regions of homology within the 5′ and 3′ ends of the GEXP07 locus were PCR amplified using the primers as outlined in Table S9. HR1 was cloned into the pUFTK plasmid containing a yeast dihydroorotate dehydrogenase cassette, which confers resistance to DSM1, by the use of the AvrII and NcoI sites, and HR2 was cloned into the SpeI and SacII site of the plasmid. The guide RNA was selected using the CHOP CHOP Web tool and cloned into BtgZI-linearized pAIO-Cas9 vector containing a human dihydrofolate reductase (hDHFR) selection cassette (60). Transfections were performed as previously described (61).

### Live-cell and immunofluorescence microscopy.

Infected red blood cell smears were fixed in an acetone and methanol solution at –20°C for 10 min. Wells were marked with a hydrophobic PAP pen and were then washed three times with PBS. Primary antibodies were diluted in 3% (wt/vol) bovine serum albumin (BSA) and were added to the wells for 1 h at room temperature and subsequently washed three times with PBS. The primary antibodies used in this study were mouse anti-REX1 (1:1,000 [20]), rabbit anti-REX1 (1:1,000 [20]), mouse anti-GFP (1:300) (Roche), rabbit anti-GFP (1:300 [62]), mouse anti-MAHRP1 (1:300 [23]), monoclonal antibody (MAb) anti-ATS (1:100 [19]), mouse anti-SBP1 (1:300 [18]), and rabbit anti-hemagglutinin (anti-HA) (1:300) (Sigma-Aldrich). Secondary anti-mouse or anti-rabbit Alexa Fluor 488, 568, or 647 antibodies (1:300 in 3% [wt/vol] BSA) (Life Technologies) were added to the wells for 1 h at room temperature, and then the wells were washed 3 times with PBS. Each well was then treated with DAPI (4′,6-diamidino-2-phenylindole) and PPD (p-phenylenediamine) antifade prior to mounting and sealing. For live-cell fluorescence microscopy, parasites were suspended in RPMI medium and mounted on a coverslip. Samples were imaged on a DeltaVision Elite restorative widefield deconvolution imaging system (GE Healthcare). Samples were excited with solid state illumination (Insight SSI; Lumencor). The following filter sets (excitation [Ex] and emission [Em] wavelengths) were used: DAPI (Ex390/18 nm, Em435/48 nm), fluorescein isothiocyanate (FITC) (Ex475/28, Em523/26), tetramethyl rhodamine isocyanate (TRITC) (Ex542/27, Em594/45), and Cy5 (Ex632/22, 676/34 nm). A 100× UPLS Apo (Olympus, 1.4 NA) oil immersion lens objective was used for imaging. For 3D structured illumination microscopy (3D-SIM), a DeltaVision OMX V4 Blaze imaging system was used (GE Healthcare). Samples were imaged using a 60× Olympus Plan APO N (1.42-numerical-aperture [NA]) oil immersion lens. The following laser emission and band pass filter sets were used: Ex488 and Em528/48 nm, Ex568 and Em609/37 or Ex642, and Em683/40. Images were processed using FIJI ImageJ software (63).

### Fluorescence image analysis.

Image quantification was performed using FIJI ImageJ software (63). Custom macros were written to analyze the mean fluorescence intensity, Maurer**’**s cleft counts, and PfEMP1 co-occurrence and fluorescence intensity. Details of these macros and the full code for each are supplied in the supplemental methods at https://doi.org/10.26188/5e4f4271c4c31 and https://doi.org/10.26188/5e4f41364567f.

### SDS-PAGE and immunoblotting of protein samples.

For immunoblotting, samples were resuspended in Bolt lithium dodecyl sulfate (LDS) sample buffer containing 50 mM dithiothreitol (DTT) and separated on Bolt 4%-to-12% Bis-Tris gel at 200 V for 32 min. Separated samples were transferred to a nitrocellulose membrane using an iBlot gel transfer device. For visualizing high-molecular-weight proteins, resuspended samples were separated on 3%-to-8% Tris-acetate gel at 150 V for 60 min and were then transferred to a polyvinylidene difluoride (PVDF) membrane at 20 V for 16 h at 4°C. Membranes were blocked in 3% (wt/vol) skim milk for 1 h at room temperature. The membranes were then probed in 3% (wt/vol) skim milk–PBS for 16 h at 4°C with the following primary antibodies: mouse anti-GFP (1:1,000; Roche), rabbit anti-SBP1 (1:1,000 [18]), MAb anti-ATS (1:100 [19]), mouse anti-REX1 (1:1,000 [20]), mouse anti-EXP1 (1:1,000), rabbit anti-spectrin (1:1,000; Sigma-Aldrich), mouse anti-MAHRP1 (1:1,000 [23]), rabbit anti-GAPDH (1:1,000), rabbit anti-HA (1:1,000; Sigma-Aldrich), and rabbit anti-HSP70-x (1:2,000 [6]). Membranes were washed 3 times in 0.05% (vol/vol) Tween 20–PBS for 10 min. Secondary antibodies (anti-mouse or anti-rabbit) conjugated to horseradish peroxidase (HRP; Promega) were diluted 1:2,000 in 3% (wt/vol) skim milk–PBS and were incubated with membranes for 1 h at room temperature. The membranes were again washed 3 times in 0.05% (vol/vol) Tween 20–PBS and then once in PBS before being incubated with Clarity ECL reagents (Bio-Rad) or SuperSignal West Femto maximum sensitivity substrate and visualized on a ChemiDoc imaging system (Bio-Rad).

### Enrichment of Maurer’s clefts.

Parasites were synchronized to a 4-h window by Percoll purification followed by sorbitol lysis. At 14 to 18 h post invasion, parasites (∼15% parasitemia) were harvested and washed in PBS. Infected red blood cells were lysed on ice with chilled hypotonic buffer (1 mM HEPES/NaOH, Roche cOmplete EDTA-free protease inhibitor, pH 7.4). The lysed infected red blood cell solution was subjected to passage through a 27-gauge needle 10 times and was then made isotonic by the addition of 4× assay buffer (200 mM HEPES-NaOH, 200 mM NaCl, 8 mM EDTA) (pH 7.4). The solution was centrifuged at 2,500 × *g* for 10 min at 4°C, and then the supernatant was collected and centrifuged again at 2,500 × *g* for 10 min at 4°C. The supernatant (containing the Maurer’s clefts) was then precleared with Pierce protein A agarose beads (Thermo Scientific) for 30 min at 4°C on a mixing wheel. The protein A agarose beads were pelleted by centrifugation, and the supernatant was incubated with GFP-Trap agarose beads (Chromotek) for 4 h at 4°C on a mixing wheel. The beads were then collected and used for downstream applications.

### Co-IP with GFP-Trap agarose beads.

Parasite-infected RBCs were purified using a VarioMACS (Miltenyi Biotec) magnet or by floatation in 70% (vol/vol) Gelofusine (Braun)–PBS. The infected RBCs were washed in RPMI medium and then solubilized on ice for 30 min in 10× pellet volumes of IP buffer (1% [vol/vol] Triton X-100, 150 mM NaCl, 50 mM Tris, 8 mM EDTA) with cOmplete protease inhibitor cocktail (Roche). The Triton X-100-insoluble material was pelleted twice by centrifugation (10 min at 16,000 × *g*). The supernatant was precleared with Pierce protein A agarose beads (Thermo Scientific) for 1 h at 4°C on a mixing wheel. The protein A beads were pelleted, and an aliquot of the supernatant was taken as the input fraction. The remainder of the supernatant was incubated with washed GFP-Trap agarose beads (Chromotek) for 16 h at 4°C. The GFP-Trap beads were then washed 5 times in IP buffer.

### Mass spectrometric analysis of Maurer’s clefts and co-IPs.

Agarose beads were washed twice in 1 mM Tris-HCl prior to elution of bound proteins. Samples were prepared for mass spectrometric analysis as previously described (31). Briefly, proteins were eluted from GFP-Trap beads by the addition of 20% (vol/vol) trifluoroethanol–formic acid (0.1%, pH 2.4) and were incubated at 50°C for 5 min. The eluate was reduced with 5 mM Tris(2-carboxyethyl)phosphine hydrochloride (TCEP) (Thermo Fisher Scientific) and neutralised with TEAB (tetraethylammonium bromide). Samples were digested with trypsin (Sigma) overnight at 37°C.

Samples were analyzed by electrospray ionization (ESI) LC-MS/MS on an Orbitrap Elite (purified Maurer’s clefts) or a Q Exactive (co-IP) mass spectrometer. Mass spectra (ProteoWizard) were searched against a custom database containing the Plasmodium falciparum 3D7 and UniProt human proteomes. Searches were performed on MASCOT (Matrix Science), with the following parameters: precursor ion mass tolerance of 10 ppm, fragment ion mass tolerance of 0.2 Da, trypsin as the cleavage enzyme, three allowed missed cleavages, and allowance for oxidation. The network map of protein interactions was created using NAViGaTOR 2.3 software, and the networks were redrawn in Adobe Illustrator.

### Infected RBC binding assay under flow conditions.

Ibidi μ-Slide 0.2 channel slides were incubated with 100 μl chondroitin sulfate A (100 μg/ml; Sigma)–1% (wt/vol) BSA–PBS overnight at 37°C. Channels were blocked with 1% (wt/vol) BSA–PBS for 1 h at room temperature and then gently flushed with warm bicarbonate-free RPMI 1640 (Invitrogen). Mid-trophozoite-stage cultures were diluted to 3% parasitemia and 1% hematocrit–bicarbonate-free RPMI 1640 and pulled through the channel at 100 μl/min for 10 min at 37°C. Unbound cells were washed out of the channel at 100 μl/min for 10 min at 37°C. Adherent cells were counted at 10 points along the axis of the channel.

### VAR2CSA ectodomain labeling and analysis via flow cytometry.

Mid-trophozoite-stage cultures were diluted to 3% parasitemia–0.4% hematocrit and plated into a 96-well plate. Cells were washed 3 times in 1% (wt/vol) BSA–PBS and incubated with a rabbit polyclonal anti-VAR2CSA antibody for 30 min at 37°C (R1945 [1:50]) (43) or a 1% (wt/vol) BSA–PBS control. Cells were washed with 1% BSA–PBS between all subsequent incubation steps, and all antibodies were diluted in 1% BSA–PBS. Cells were incubated with mouse anti-rabbit IgG (Dako; 1:100) for 30 min at 37°C and then with mouse anti-GFP (Alexa Fluor 488) under the same conditions. Following tertiary labeling, the cells were washed 3 times in 1% (wt/vol) BSA–PBS. Nuclei were stained with SYTO-61 as described previously (69) and analyzed via the use of a flow cytometer (BD FACSCanto II). SYTO-61-positive cells were gated, and the levels of Alexa Fluor 488 fluorescence intensity were measured. The plotted data represent the levels of mean fluorescence intensity (MFI) of the doubly positive population minus the background MFI.

### Trypsin cleavage of surface-exposed *Pf*EMP1.

Late-stage-infected RBCs were purified and incubated with 20 volumes of 1 mg/ml tosylsulfonyl phenylalanyl chloromethyl ketone (TPCK)-treated trypsin–PBS (Sigma) or with trypsin and soybean trypsin inhibitor (5 mg/ml; Sigma) at 37°C for 1 h. Trypsin activity was quenched with a 15-min incubation in trypsin inhibitor at room temperature for 15 min. The cells were subsequently solubilized in 10 volumes of 1% Triton X-100–PBS containing 1× cOmplete protease inhibitor (Roche) and incubated on ice for 30 min. The sample was centrifuged at 16,000 × *g* at 4°C for 10 min, and the resulting pellet was washed in the Triton solution 2 additional times. The resulting Triton-insoluble pellet was solubilized in 20 volumes of 2% SDS–PBS and mixed on a rotating wheel for 30 min. This SDS-soluble fraction was then subjected to separation by SDS-PAGE and transferred to a PVDF membrane for Western blotting.

### Parasite growth assay.

Parental CS2 and ΔGEXP07 cell lines were synchronized to a 2-h window and diluted to 1.5% late-stage parasitemia. Every 48 h, infected red blood cells were taken from the culture and stained with SYTO-61 nuclear stain and parasitemia was calculated by flow cytometry as previously described (69). The cultures were then diluted by the same dilution factor. Parasitemia was recorded over 4 cycles, and the data were normalized to the parental CS2 line.

### Sorbitol sensitivity assay.

Mid-trophozoite-stage-infected red blood cells at 2% to 5% parasitemia were incubated at 37°C for 10 min with 1 of 12 solutions with between 0% and 5.5% (wt/vol) d-sorbitol. The level of parasitemia present following treatment was measured via flow cytometry following incubation with nuclear stain SYTO-61, and 30,000 to 50,000 events were recorded per well as previously described (69). Parasitemia was normalized to an internal RPMI medium control for each experiment. The data were then used to calculate the EC_50_ values for each separate experiment. The EC_50_ values from each experiment are plotted.

### Microbead filtration.

Spleen mimic filtration was performed to assess the deformability properties of parasite-infected RBCs (64). Synchronous parasites present at 22 h post invasion were prepared at 6% parasitemia and 1% hematocrit in 1% (wt/vol) AlbuMAX II–1× PBS and pumped through the microbead bed using a syringe pump at a flow rate of 60 ml/min. The data presented represent percentages of parasites present in the flowthrough relative to starting parasitemia. The means and standard errors are plotted from 3 separate experiments. A Student's *t* test was used to evaluate statistical significance.

### Membrane shearing.

Glass coverslips were cleaned with acetone and 50% methanol prior to treatment with 3-aminopropyl triethoxysilane (APTES) and bis-sulfosuccimidyl suberate and addition of the ligand erythroagglutinating phytohemagglutinin (PHAE) as previously described (65). Infected RBCs were immobilized on the functionalized glass slides and were then sheared by applying a hypotonic buffer (5 mM Na_2_HPO_4_/NaH_2_PO_4_, 10 mM NaCl, pH 8) from a 30-ml syringe (23-gauge needle) at an angle of ∼20° (66). The membrane disks were placed in PBS prior to downstream imaging.

### Scanning electron microscopy.

Whole infected RBC cell samples bound to glass coverslips were immersed in 0.05% glutaraldehyde–PBS for 20 min prior to fixation in 2.5% glutaraldehyde for 2 h at room temperature. Sheared membranes were prepared for scanning electron microscopy as previously described (2). In brief, sheared cells were fixed immediately after lysis with 2.5% glutaraldehyde–PBS for 2 h at room temperature. Both whole-cell samples and sheared-cell samples were then washed thoroughly with deionized water and dehydrated via sequential 5-min incubations in 20%, 50%, 70%, 80%, 90%, 95%, and (3×) 100% ethanol followed by critical point drying in a Leica CPD300 critical point dryer. Samples were stored under desiccation conditions and were coated with gold immediately before imaging. Coverslips were coated with gold at 25 mA for 40 s and 75 s using a Dynavac sputter coating instrument to thicknesses of ∼0.2 nm and ∼0.4 nm for the sheared and whole cells, respectively. Images were acquired with the Everhart-Thornley detector of an FEI Teneo SEM in Optiplan mode using at a working distance of 5 mm, a beam current level of 50 pA, and 2 kV accelerating voltage.

### Immunoelectron tomography.

Infected red blood cells (20 to 30 h post invasion) were magnet purified, washed in PBS, and fixed in 10 pellet volumes of 2% (vol/vol) paraformaldehyde (PFA)–PBS for 20 min at room temperature. Cells were washed and then permeabilized in 10 pellet volumes of PBS with 1 hemolytic unit (HU) of equinatoxin II for 6 min. After the washing step, the cells were fixed again in 2% PFA–PBS for 5 min, washed, and then blocked in suspension for 1 h with 3% BSA–PBS. The cells were incubated with anti-REX1 (1:50) for 2 h, washed, and then incubated with a gold secondary antibody for 1 h (1:15; Aurion protein A electron microscopy [EM]-grade 6-nm-diameter gold; catalog no. JA806-111). Cells were washed in 3% (wt/vol) BSA–PBS and then in PBS to remove the BSA.

Cell pellets were resuspended and fixed in 2.5% glutaraldehyde at 4°C for at least 1 h, prior to preembedding in low-melting-point agarose. Cells were then post fixed in 1% potassium ferricyanide {K_3_[Fe(CN)_6_]}-reduced osmium tetroxide (OsO_4_) solution for 1 h. Blocks were then rinsed five times for 3 min each time in double-distilled water (ddH_2_O) and then dehydrated by sequential incubation (for 5 min at each step) in 20%, 50%, 70%, 80%, 90%, 95%, and 100% ethanol. Blocks were incubated in 100% ethanol twice more for 5 min each time followed by ethanol/acetone solution (1:1) incubation for 30 min and then 100% acetone incubation for 30 min. Acetone was substituted for acetone–1% thiocarbohydrazide resin (1:1) for 2 h, and the reaction mixture was infiltrated with 100% resin twice for 12 h each time and polymerized in an oven at 60°C.

Resin blocks were trimmed and sectioned on an ultramicrotome (Leica EM UC7; Leica Microsystems, Wetzlar, Germany), and 70-nm and 300-nm sections were prepared for imaging and electron tomography, respectively. The sections were then subjected to post staining by the use of 4% uranyl acetate–water for 10 min and were citrated with Reynold’s lead for 10 min. The 300-nm sections were overlaid with 10-nm-diameter fiducial gold particles on both sides of the grid. Imaging and electron tomography were performed on an FEI Tecnai F30 electron microscope (FEI Company, Hillsboro, OR) at an accelerating voltage of 300 kV. The tilt series were acquired for every 2° in the range between −70° and 70° tilts. Virtual sections were reconstructed from the raw tilt series in IMOD using a weighted back-projection algorithm (67).

### Data accessibility.

The mass spectrometry proteomics data have been deposited to the ProteomeXchange Consortium via the PRIDE (68) partner repository with the data set identifier PXD014873.
