# Supplementary figures and images for: Role of Plasmodium falciparum Protein GEXP07 in Maurer’s Cleft Morphology, Knob Architecture, and P. falciparum EMP1 Trafficking
Source: mBio. 2020 Mar 17;11(2):e03320-19. doi: 10.1128/mBio.03320-19 (PMC7078486; doi:10.1128/mBio.03320-19)

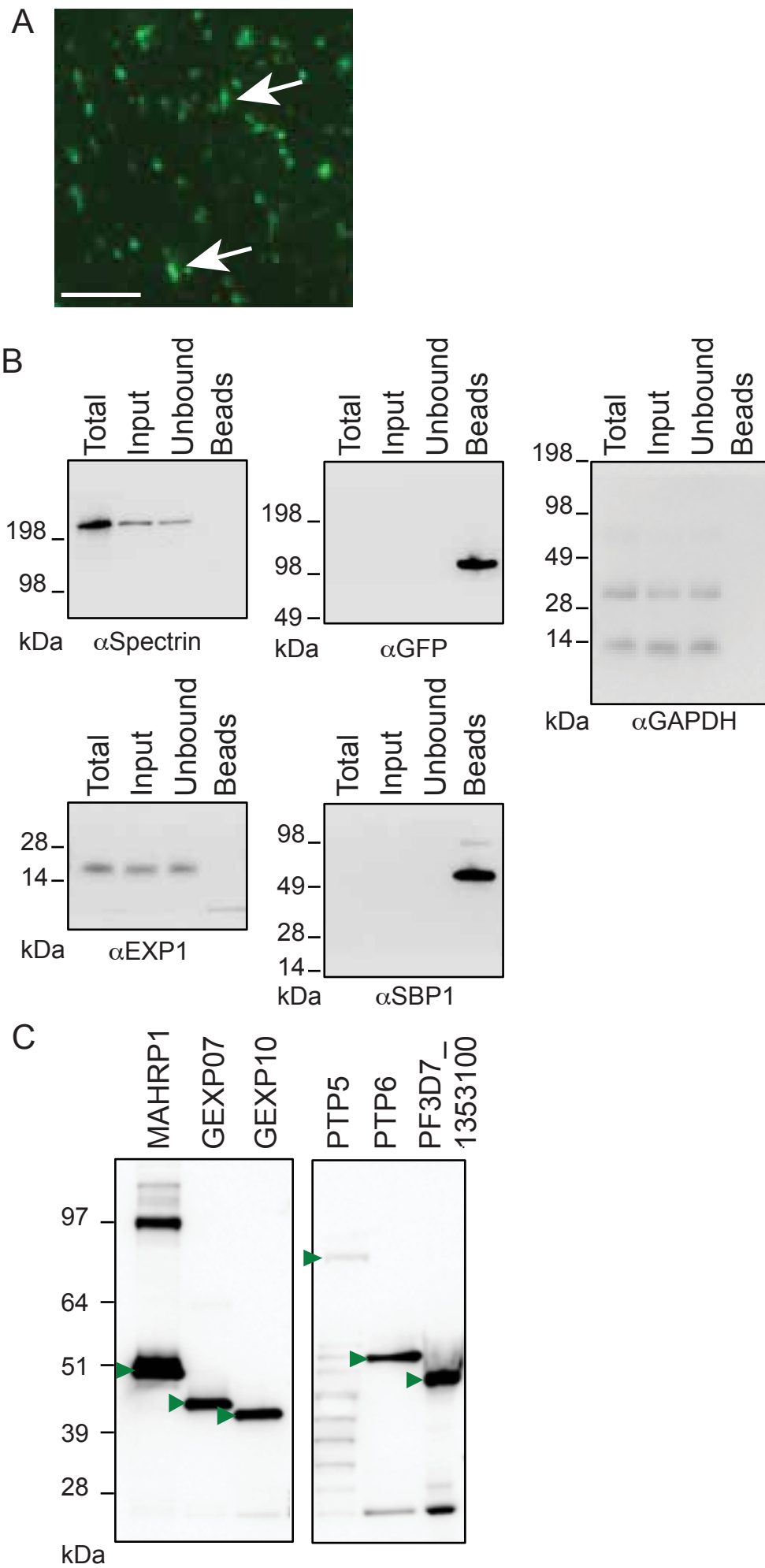

Fig S1

Supplement: FIG S1 [file mBio.03320-19-sf001.pdf]

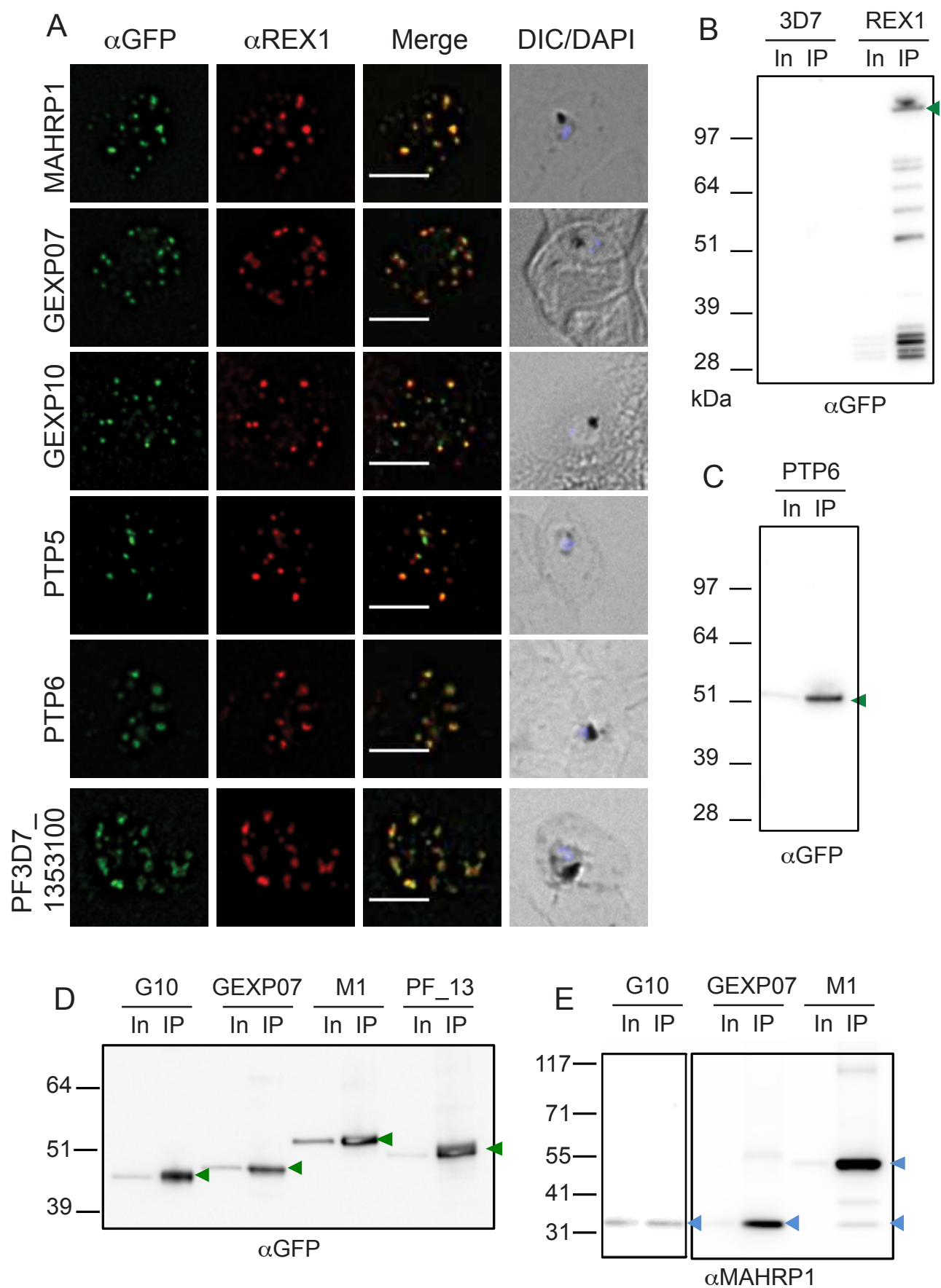

Fig S2

Supplement: FIG S2 [file mBio.03320-19-sf002.pdf]

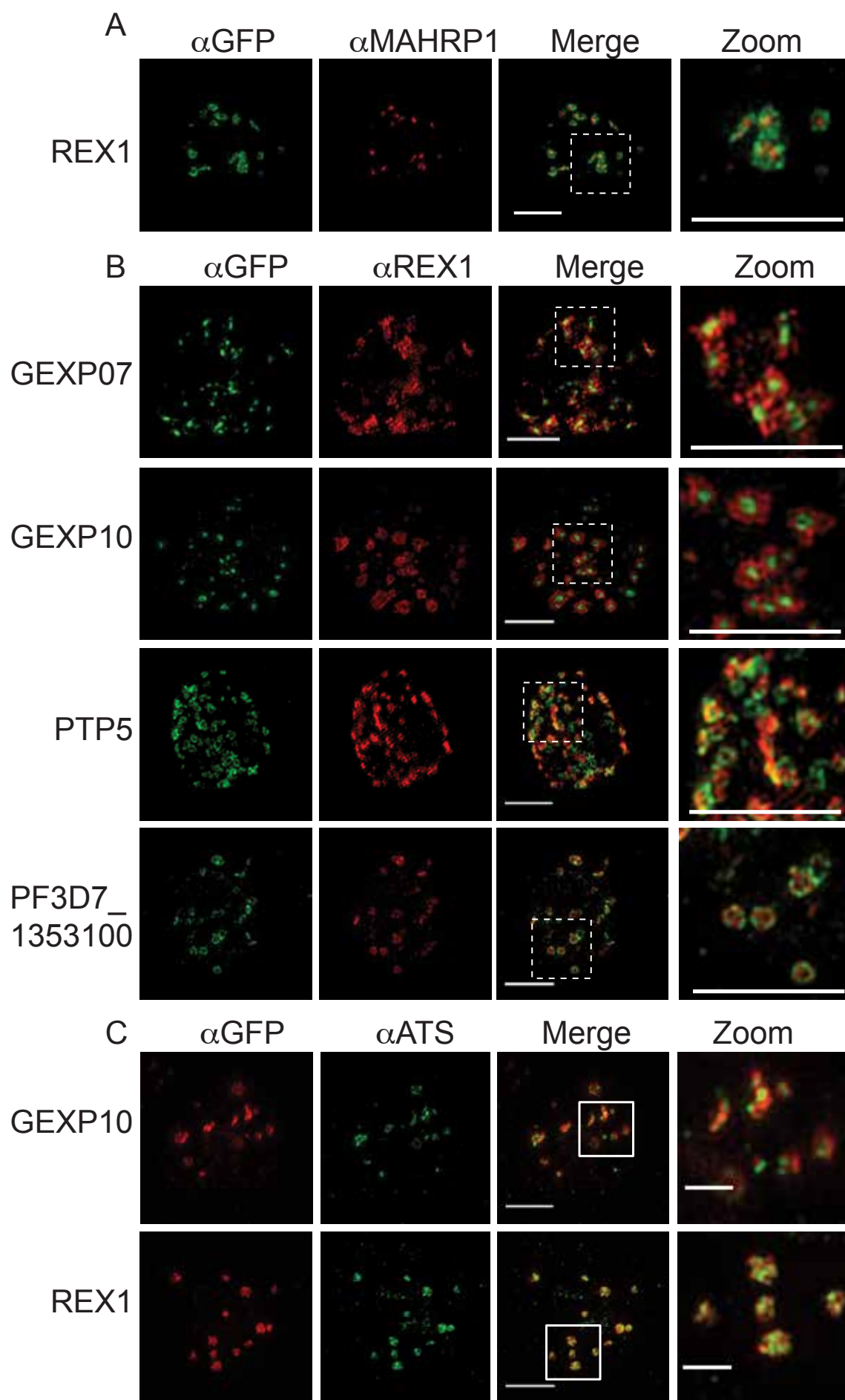

Fig S4

Supplement: FIG S4 [file mBio.03320-19-sf004.pdf]

A

# Gene Disruption Strategy

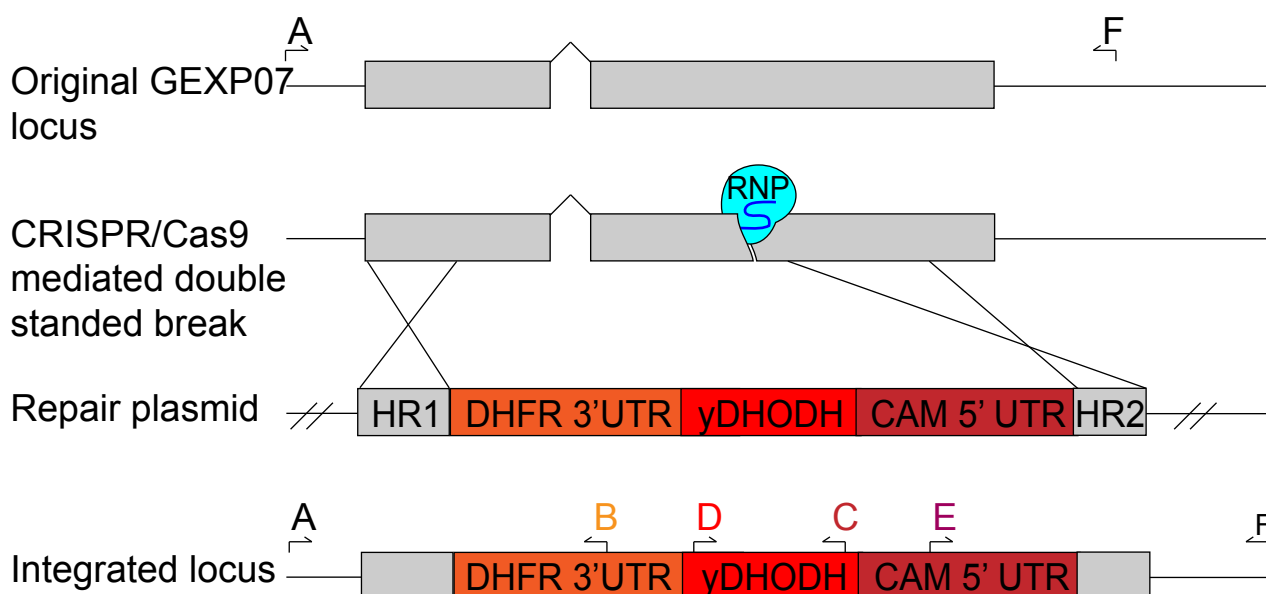

B

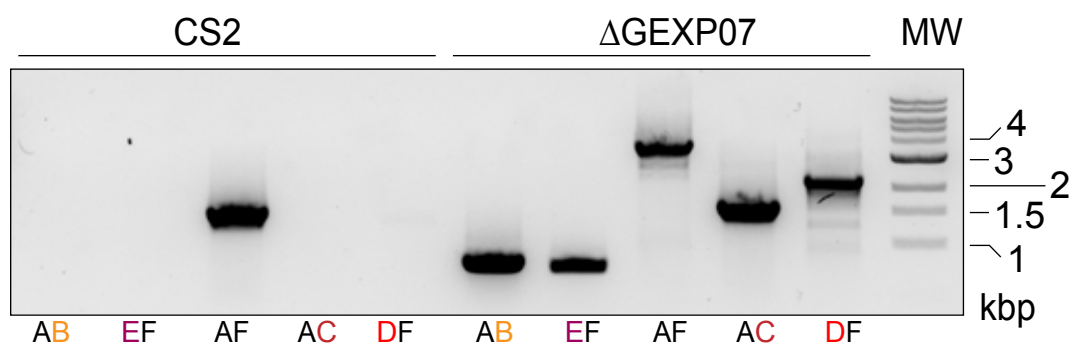

Fig S5

Supplement: FIG S5 [file mBio.03320-19-sf005.pdf]

**A CS2**

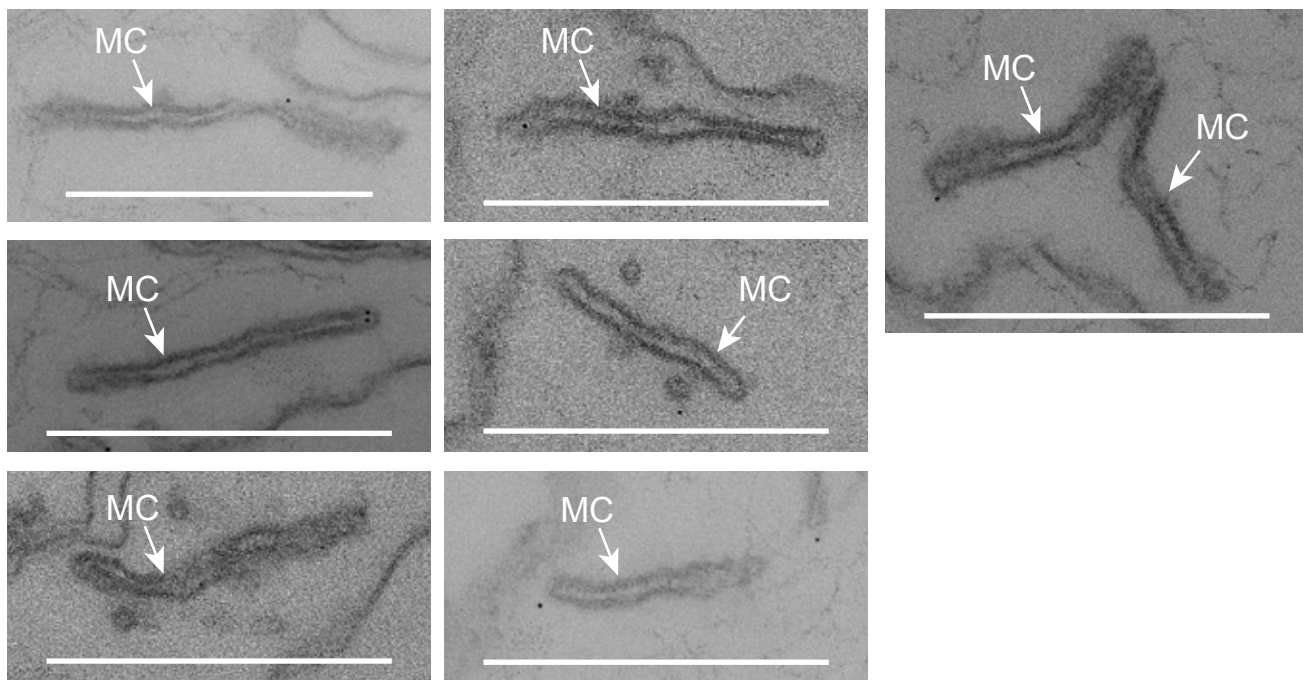

**B  $\Delta$ GEXP07**

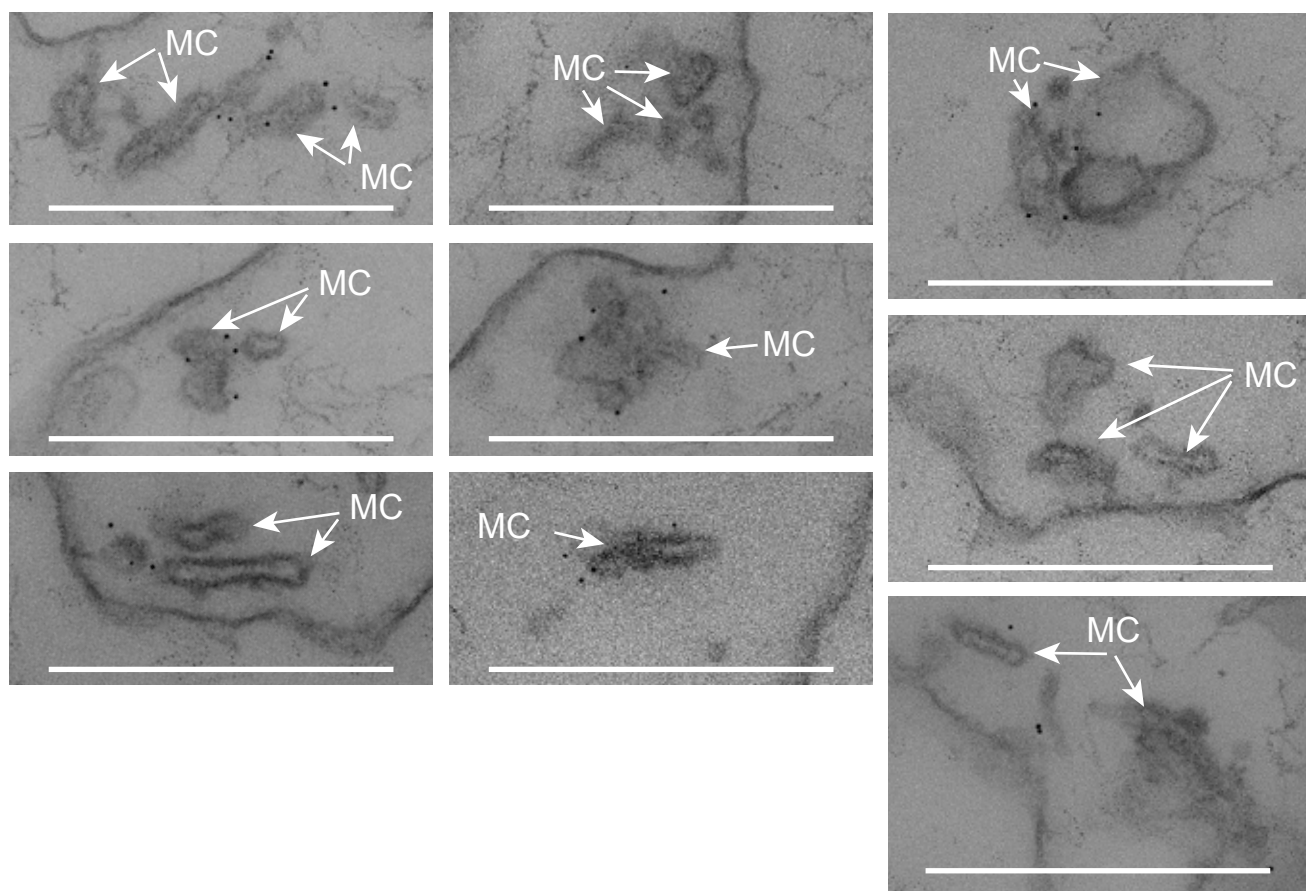

**Fig S6**

Supplement: FIG S6 [file mBio.03320-19-sf006.pdf]

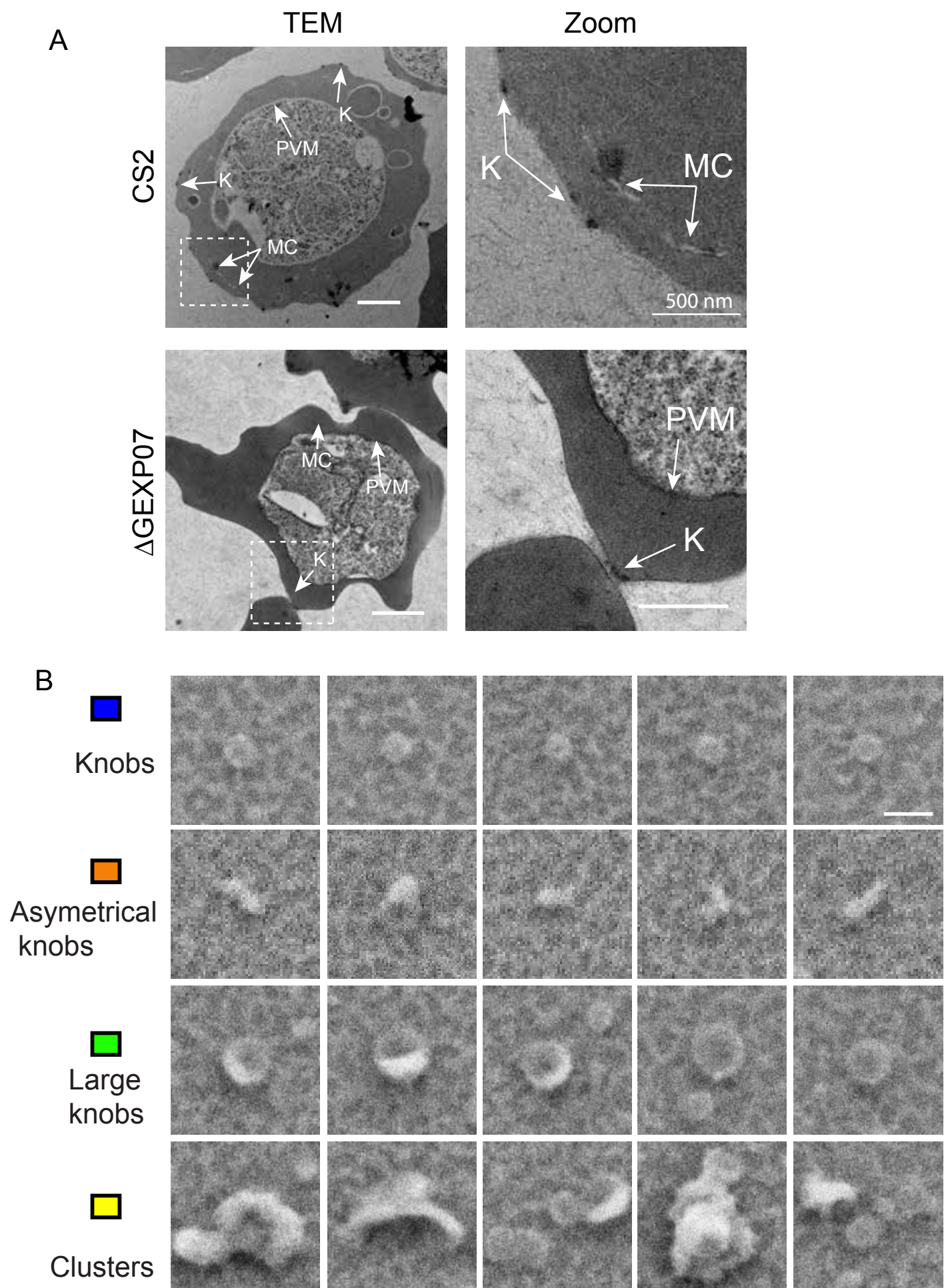

Fig S7

Supplement: FIG S7 [file mBio.03320-19-sf007.pdf]
