# Supplementary material for: Role of Plasmodium falciparum Protein GEXP07 in Maurer’s Cleft Morphology, Knob Architecture, and P. falciparum EMP1 Trafficking
Source: mBio. 2020 Mar 17;11(2):e03320-19. doi: 10.1128/mBio.03320-19 (PMC7078486; doi:10.1128/mBio.03320-19)
Supplement: FIG S3 [file mBio.03320-19-sf003.pdf]

A

Network map of protein connectivity

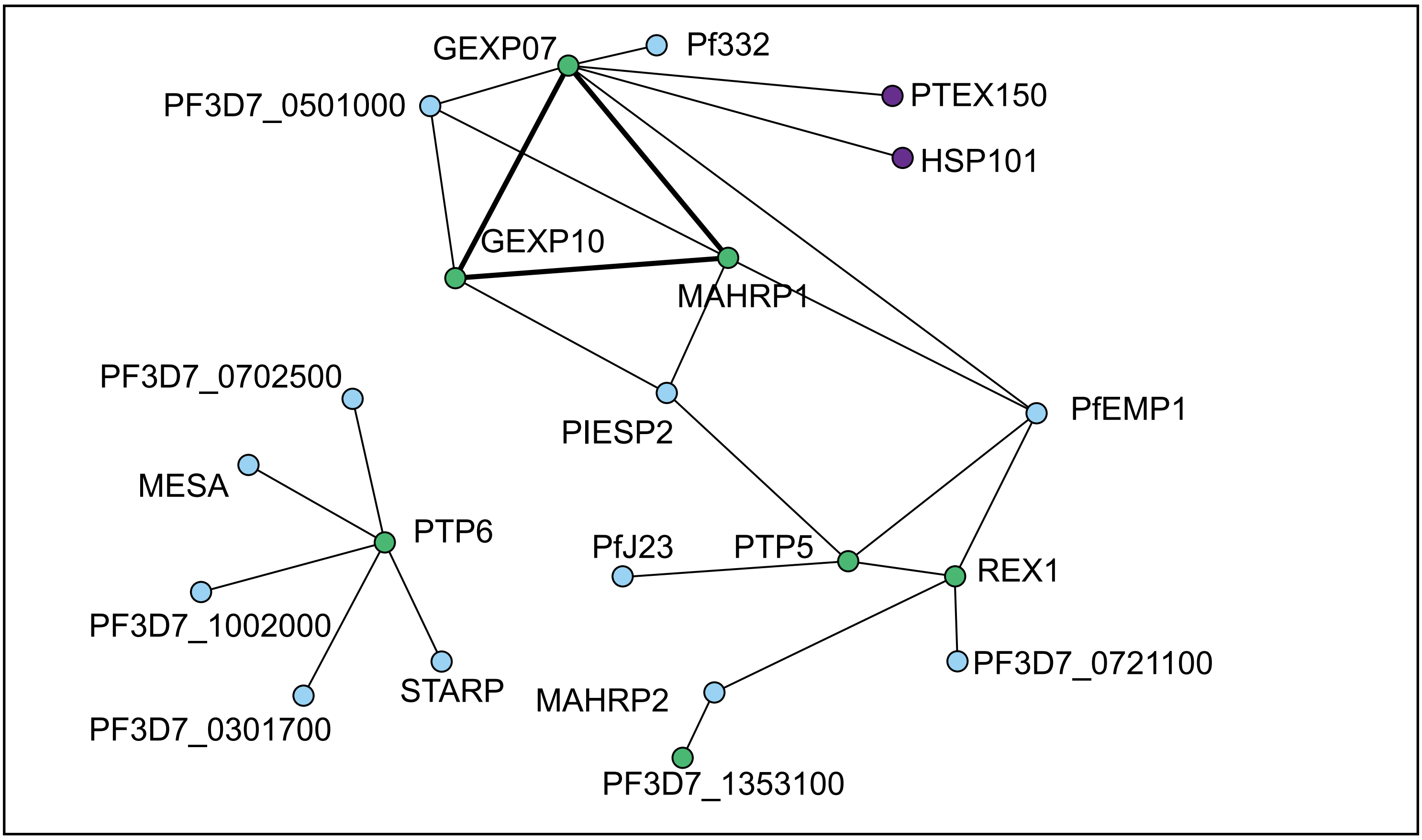

B

Network map of protein connectivity including data from the literature

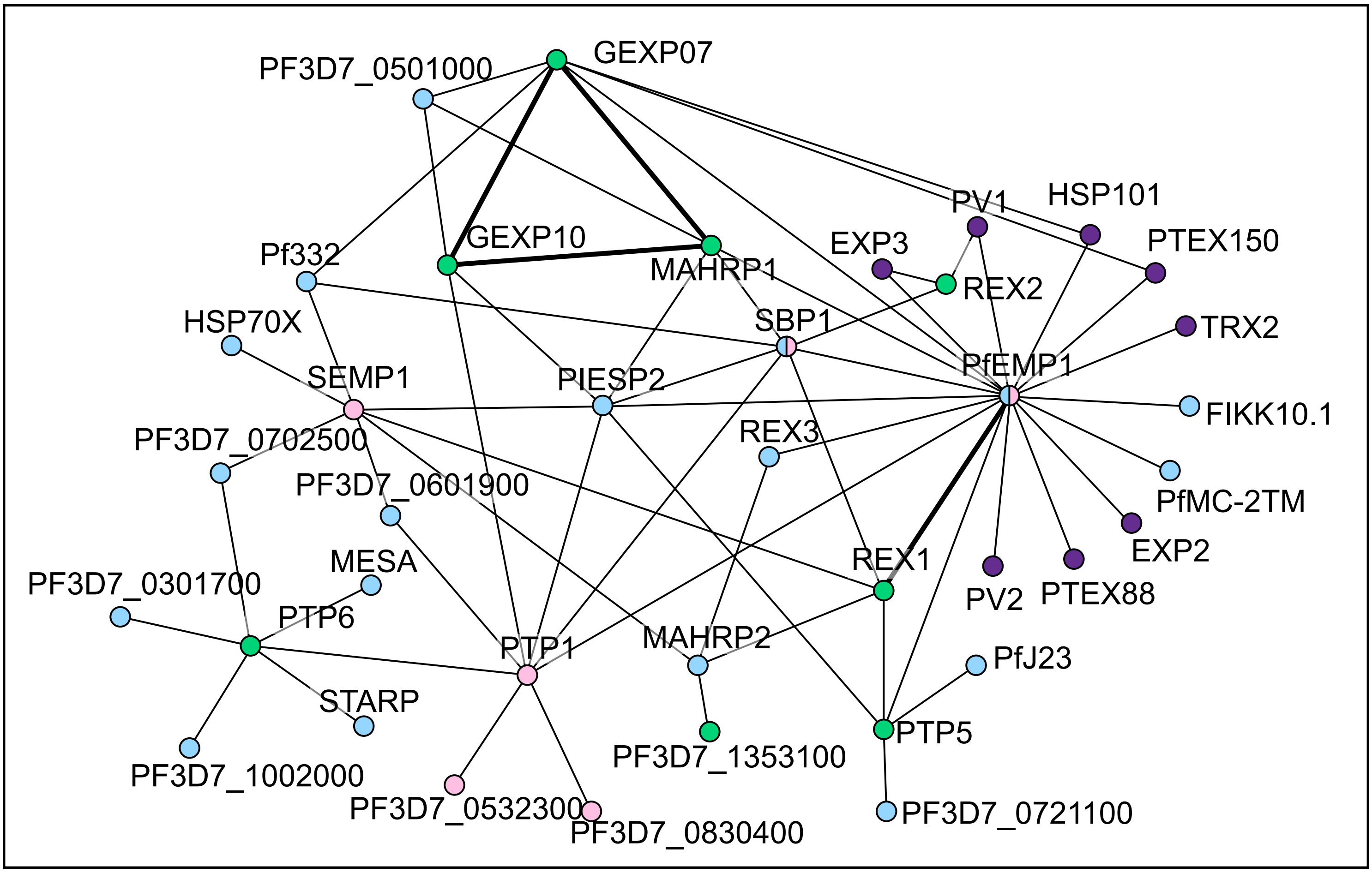

Fig S3
